# Empirical comparison of analytical approaches for identifying molecular HIV-1 clusters

**DOI:** 10.1038/s41598-020-75560-1

**Published:** 2020-10-29

**Authors:** Vlad Novitsky, Jon A. Steingrimsson, Mark Howison, Fizza S. Gillani, Yuanning Li, Akarsh Manne, John Fulton, Matthew Spence, Zoanne Parillo, Theodore Marak, Philip A. Chan, Thomas Bertrand, Utpala Bandy, Nicole Alexander-Scott, Casey W. Dunn, Joseph Hogan, Rami Kantor

**Affiliations:** 1 Brown University, Providence, RI USA; 2 Research Improving People’s Life, Providence, RI USA; 3 Yale University, New Haven, CT USA; 4 Rhode Island Department of Health, Providence, RI USA

**Keywords:** Diseases, Molecular medicine

## Abstract

Public health interventions guided by clustering of HIV-1 molecular sequences may be impacted by choices of analytical approaches. We identified commonly-used clustering analytical approaches, applied them to 1886 HIV-1 Rhode Island sequences from 2004–2018, and compared concordance in identifying molecular HIV-1 clusters within and between approaches. We used strict (topological support ≥ 0.95; distance 0.015 substitutions/site) and relaxed (topological support 0.80–0.95; distance 0.030–0.045 substitutions/site) thresholds to reflect different epidemiological scenarios. We found that clustering differed by method and threshold and depended more on distance than topological support thresholds. Clustering concordance analyses demonstrated some differences across analytical approaches, with RAxML having the highest (91%) mean summary percent concordance when strict thresholds were applied, and three (RAxML-, FastTree regular bootstrap- and IQ-Tree regular bootstrap-based) analytical approaches having the highest (86%) mean summary percent concordance when relaxed thresholds were applied. We conclude that different analytical approaches can yield diverse HIV-1 clustering outcomes and may need to be differentially used in diverse public health scenarios. Recognizing the variability and limitations of commonly-used methods in cluster identification is important for guiding clustering-triggered interventions to disrupt new transmissions and end the HIV epidemic.

## Introduction

Prevention of new HIV transmissions remains a major challenge in the global HIV epidemic, and innovative methods are needed to disrupt them^1^. Inferences about HIV transmission networks could guide public health interventions and assist in design of prevention strategies^2–4^. Despite recent advances in HIV research, including more robust and affordable viral sequencing and sophisticated bioinformatic pipelines, information about the structure and dynamics of HIV transmission networks, and how they should inform public health interventions, remains poorly understood.

HIV-1 *pol* sequences obtained through routine clinical drug resistance testing have been used successfully to identify molecular clusters, characterize epidemics, and disrupt outbreaks^2,3,5^. Accurate identification and monitoring of molecular HIV clusters may improve understanding of HIV transmission networks and the underlying mechanisms of virus spread, and are integrated into the four pillars towards ending the US HIV epidemic^2,6^.

The definition and determination of a “molecular HIV cluster” depends on statistical methods, the software tools used to implement those methods, analysis parameters, and thresholds used to interpret results (see literature review in Supplementary Materials). Heterogeneity of analytical approaches, dependence on parameters such as “threshold” and the interpretation of clustering results make it difficult to compare results between and across studies, or to discern whether and how the choice of method impacts clustering results. Limited comparisons of methods for HIV clustering have illustrated variability^7–17^, although justification for any specific method or a systematic comparison between methods are limited. It remains unclear whether specific methods should be applied uniformly across different public health and epidemic scenarios, or whether they should be tailored to specific settings or goals. In the context of ending the HIV epidemic^6^, addressing these research gaps may improve HIV cluster analysis and its real-time incorporation into public health interventions to disrupt HIV transmissions.

Knowing how distinct analytical approaches identify molecular HIV clusters under different parameterizations can be used to select one or more of those methods for public health surveillance and outreach. In this study, we performed a literature review, selected commonly-used approaches to identify molecular HIV-1 clusters, and assessed the sensitivity of cluster structure to method and threshold selection. Determining within- and between- method concordance across different parameterizations and thresholds leads to better understanding of their strengths and limitations, and ultimately may be used to improve public health efforts to prevent new HIV transmissions.

## Results

### Sensitivity to topological support and distance thresholds within analytical approaches

**Figure 1:**
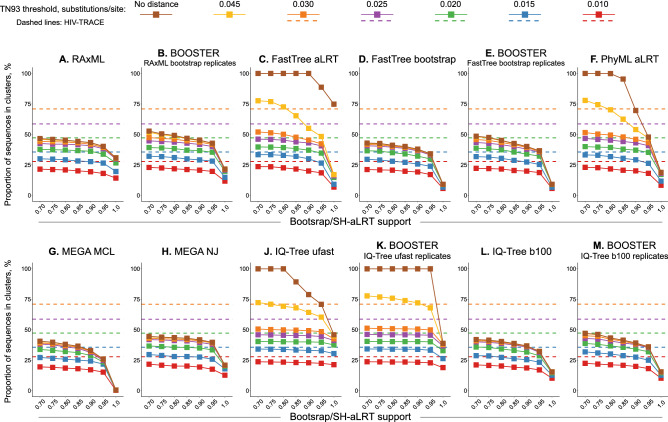
Comparison of proportion of HIV-1 sequences in clusters within commonly-used analytical approaches. Graphs A-M each represent the 12 model-based methods/variations examined. Solid lines in each graph represent the range of proportions of clustered sequences (Y axis) according to topological support (X axis) and distance thresholds (colored squares (legend at the top of the Figure), matching the line colors). Color-matching dashed lines in each graph represent the range of proportions of clustered sequences identified by HIV-TRACE according to five distance thresholds (see text for details).

Figure 1 shows, for each method and for a range of 49 topological support and TN93-distance thresholds, the *proportion* of sequences that are in clusters. Within specific methods, there is little difference across topological supports at the lower end of the scales. At topological support of 0.90 and more prominently 0.95, clustering proportion was lower for most methods, except for (a) IQ-Tree ultrafast bootstrap (Fig. 1J) at distance threshold ≤ 0.025 substitutions/site, and (b) its related BOOSTER version (Fig. 1K). The effect of distance threshold was more pronounced over the range of distance thresholds 0.010‒0.045 substitutions/site, with lower clustering proportion at lower distance thresholds. Without using any distance threshold, some methods placed all sequences in a single or very few clusters [e.g., low topological support in FastTree aLRT (Fig. 1C), PhyML aLRT (1F) ultra-fast IQ-Tree BOOSTER (1K), and ultra-fast IQ-Tree (1J)]. This is because strong topological support for deep nodes in the phylogeny can create artificial large clusters that include most tips, if distance thresholds do not exclude these deeper nodes from cluster identification. As compared to HIV-TRACE, the model-based methods had lower proportions of clustered sequences at compatible levels of distance thresholds (solid and dashed lines of the same color in Fig. 1). Similar patterns were observed for the *number* of identified clusters (Supplementary Figure S3).

To test the robustness of these phylogenetic reconstructions and examine whether results vary across runs we used RAxML as an example. We conducted 100 repeated runs (1000 bootstrap replications each) using different seed for each run, and from each run inferred proportions of viral sequences in clusters for the 49 combinations of topological support and pairwise distances. We found extremely low variance (median 0.022%; IQR 0.011–0.057%; range 0.006–0.398%) across repeated runs, supporting robustness of findings.

The effect of cluster definition criteria on the size of individual clusters was minimal across the 49 combinations of topological support and pairwise distances using RAxML (Supplementary Figure S4). Cluster size distributions demonstrated similar shape patterns with many small clusters and few large clusters throughout all cluster definition criteria.

### Comparison of clustering patterns between analytical approaches

**Figure 2:**
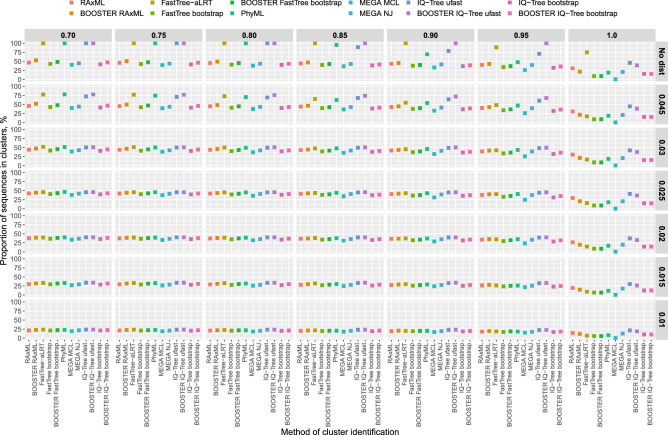
Comparison of proportion of HIV-1 sequences in clusters between commonly-used analytical approaches. Each of the 49 panels demonstrates proportions of HIV sequences in clusters (Y axis) identified by the 12 selected methods (X axis; also represented by colors and outlined in the legend above the panels), representing a distinct combination of topological support (outlined in the gray line above the panels) and distance thresholds (outlined in the gray line to the right of the panels); see text for more details.

Examination of sequence clustering patterns between the 12 model-based methods by the 49 topological support and distance threshold combinations revealed two distinct patterns (Fig. 2). For the most stringent topological support (rightmost column) and the most relaxed distance thresholds (two top rows), there is considerable variation in clustering across the selected methods. In contrast, relaxed topological support combined with intermediate or stringent distance thresholds demonstrated noticeable similarity in clustering across the selected methods. Similar patterns were found for the *number* of identified clusters (Supplementary Figure S5).

### Differences between method pairs in proportions of clustered sequences

Proportions of clustered sequences in each of the seven model-based methods plus HIV-TRACE, according to both strict and relaxed thresholds, are summarized in Table 1. The proportion of sequences that clustered ranged from 22% (MEGA; 156 clusters) to 30% (IQ-Tree ultrafast; 187 clusters) with strict thresholds, and 38% (MEGA; 223 clusters) to 54% (PhyML aLRT; 234 clusters) with relaxed thresholds. Using HIV-TRACE with a threshold of 0·015 substitutions/site, 36% of viral sequences were found in 172 clusters.

**Table 1:** HIV-1 clusters identified by seven commonly-used analytical approaches according to strict and relaxed sets of topological support and distance thresholds. HIV-TRACE at 0.015 TN93 distance threshold identified 172 clusters (671 sequences in clusters; 36%).

Methods	Strict thresholds			Relaxed thresholds		
	Topological support	Mean TN93 pairwise distances	# Of clusters (# of sequences in clusters; %)	Topological support	Mean TN93 pairwise distances	# Of clusters (# of sequences in clusters; %)
RAxML	0.95; rapid bootstrap; 1000 replicates	0.015	167 (500; 27%)	0.80; rapid bootstrap; 1000 replicates	0.045	220 (847; 45%)
FastTree aLRT	0.95; aLRT	0.015	163 (500; 27%)	0.90; aLRT	0.030	212 (856; 45%)
FastTree bootstrap	0.95; regular bootstrap; 1000 replicates	0.015	146 (451; 24%)	0.80; rapid bootstrap; 1000 replicates	0.045	201 (772; 41%)
PhyML aLRT	0.95; aLRT	0.015	162 (496; 26%)	0.90; aLRT	0.045	234 (1019; 54%)
MEGA	MCL; 0.95; regular bootstrap; 1000 replicates	0.015	156 (411; 22%)	MCL; 0.80; regular bootstrap; 1000 replicates	0.045	223 (712; 38%)
IQ-Tree ufast	1.0; ultrafast bootstrap; 1000 replicates	0.015	187 (573; 30%)	0.95; ultrafast bootstrap; 1000 replicates	0.030	231 (913; 48%)
IQ-Tree regular	0.95; regular bootstrap; 100 replicates	0.015	146 (439; 23%)	0.80; regular bootstrap; 100 replicates	0.045	198 (758; 40%)

*aLRT *approximate likelihood ratio test,* MCL *maximum composite likelihood, *ufast* ultrafast bootstrap, # number.

**Figure 3:**
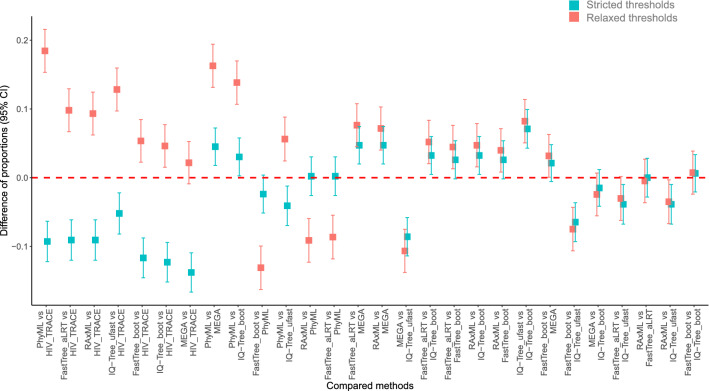
Differences of proportions of clustered HIV-1 sequences between method-pairs. The graph represents differences in proportions of clustered HIV-1 sequences (Y axis; shown with 95% CI) that were identified by pairs of the seven methods (X axis). Differences are ranked from left to right in descending order of absolute values, according to relaxed (red squares) and strict (green squares) thresholds. The red dashed line outlines a proportion difference of zero. Positive or negative differences in proportions depend on the directionality of the comparison between each methods-pair; see text for more details.

Differences in proportions of clustered sequences (and 95% confidence interval (CI)) between method-pairs using strict and relaxed thresholds are presented in Fig. 3 (in descending order of differences) and Supplementary Table S2; these CIs are not adjusted for multiple comparisons. The differences ranged from − 14 to 7% for the strict thresholds (largest being 14% between MEGA and HIV-TRACE) and from − 13 to 18% for the relaxed thresholds (largest being 18% between PhyML and HIV-TRACE). Differences in proportions of clustered sequences between model-based methods and HIV-TRACE (seven leftmost comparisons in Fig. 3) had negative values for strict thresholds and positive values for relaxed thresholds. For strict thresholds, HIV-TRACE clustered between 9 and 14% (mean 10%) more sequences than model-based methods. In contrast, for relaxed thresholds, the proportion of sequences placed in a cluster by HIV-TRACE was between 1 and 18% (mean 9%) lower than for model-based methods.

### Concordance of clustering: identifying similar sequence pairs in the same clusters

**Figure 4:**
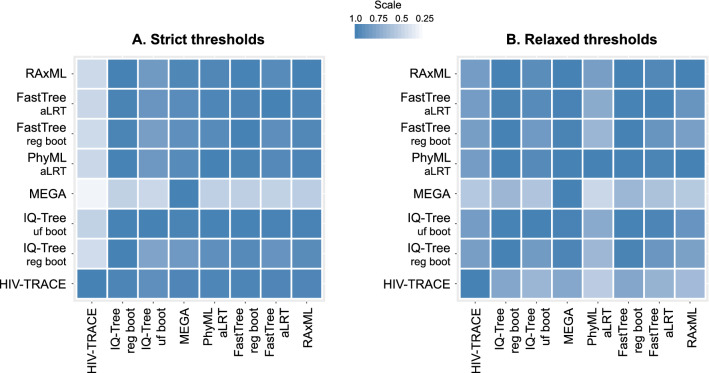
Concordance of HIV-1 clustering: proportion of sequence pairs clustered by method-pairs. In these asymmetric heatmaps, each of the 64 small squares in each panel represents the proportion of sequence pairs that were clustered together in one of the eight methods examined (listed at the bottom of the heatmap), and also in the second paired method (listed on the left of the heatmap). For example, the 3rd square from the left in the top row shows proportion of sequence pairs that clustered together by IQ-Tree ultra-fast bootstrap that also clustered together by RAxML; with the denominator being the proportion of clustered sequence pairs in IQ-Tree ultra-fast bootstrap analysis). The squares on the diagonal line from bottom left to upper right of each panel show concordance between the same methods, which is always 100%. Panel A demonstrates analyses according to strict thresholds and panel B according to relaxed thresholds (for more methods and thresholds details see text and Table 1). The scale of proportions for both panels is also shown.

We examined concordance of the seven model-based methods and HIV-TRACE in identifying the same sequence pairs in the same clusters (Fig. 4; asymmetrical heatmaps for strict and relaxed thresholds; Supplementary Tables S3‒S4). At the strict set of thresholds (Fig. 4A; Supplementary Table S3) the *median percent concordance* (proportion of pairs of sequences that are clustered by both method-pairs) was 93% (IQR 78‒98%; range 17‒100%). Two noticeable exceptions were (a) MEGA (fifth horizontal line) that shared only 38‒45% of clustered sequence pairs identified by other model-based methods, and only 17% of pairs identified by HIV-TRACE; and (b) HIV-TRACE (most left vertical line)—which shared between 17 and 41% of sequence pairs that were detected by other methods. At the strict thresholds, RAxML demonstrated the highest *mean summary percent concordance* (88%), and HIV-TRACE the lowest (65%).

At the relaxed set of thresholds (Fig. 4B; Supplementary Table S4), the *median percent concordance* was 82% (IQR 69‒99%; range 38‒100%). MEGA demonstrated better concordance than in the strict set, 38‒61%. PhyML aLRT detected ≥ 94% of sequence pairs clustered in other model-based methods (80% of sequence pairs identified in clusters by HIV-TRACE). However, only 38‒78% of sequence pairs that clustered in PhyML aLRT were found in clusters by other methods. FastTree regular bootstrap demonstrated the highest *mean summary percent concordance* (87%), while MEGA (73%) was the lowest among model-based methods. Overall, the range of *mean summary percent concordance* between analyzed methods was 65–88% for the strict thresholds and 69‒87% for the relaxed thresholds.

### Concordance of clustering: Identifying identical clusters

**Figure 5:**
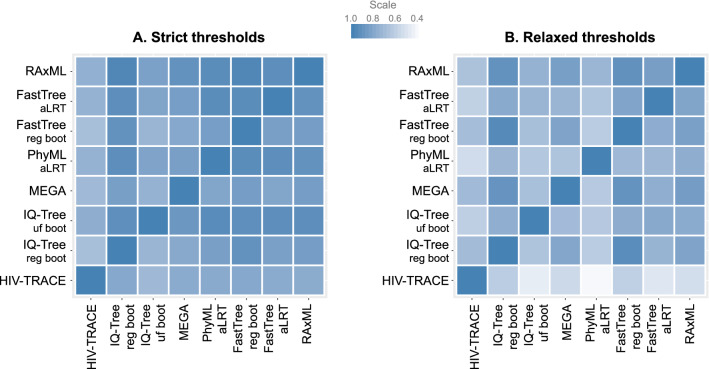
Concordance of HIV-1 clustering: proportion of identical clusters in method-pairs. In these asymmetric heatmaps, each of the 64 small squares in each panel represents the proportion of identical clusters that were identified in one of the eight methods examined (listed at the bottom of the heatmap), and also in the second paired method (listed on the left of the heatmap). The squares on the diagonal line from bottom left to upper right of each panel show concordance between the same methods, which is always 100%. Panel A demonstrates analyses according to strict thresholds and panel B according to relaxed thresholds (for more methods and thresholds details see text and Table 1). The scale of proportions for both panels is also shown.

At the strict thresholds the *median percent concordance* in identifying identical clusters in all pairwise comparisons of methods was 84% (IQR 78‒92%; range 67‒97%) (Fig. 5A; Supplementary Table S5). MEGA was on the lower end of identifying identical clusters (70‒85%). RAxML demonstrated the highest *mean summary percent concordance* (88%), and MEGA (82%) the lowest among model-based methods. At the relaxed set of thresholds (Fig. 5B; Supplementary Table S6), *median percent concordance* was 72% (IQR 63‒80%; range 38‒95%). RAxML had the highest *mean summary percent concordance* (79%), while PhyML aLRT (64%) had the lowest among model-based methods. Overall, the range of *mean summary percent concordance* between analyzed methods in this analysis was 75‒88% for the strict thresholds, and 57‒79% for the relaxed thresholds.

### Concordance of clustering: identifying non-clustered sequences

**Figure 6:**
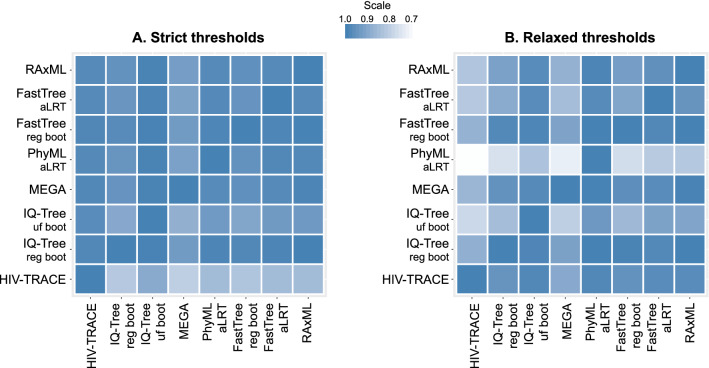
Concordance of HIV-1 clustering: proportion of sequences not clustered by method-pairs. In these asymmetric heatmaps each of the 64 small squares in each panel represents the proportion of non-clustered sequences that were identified in one of the eight methods examined (listed at the bottom of the heatmap), and also in the second paired method (listed on the left of the heatmap). The squares on the diagonal line from bottom left to upper right of each panel show concordance between the same methods, which is always 100%. Panel A demonstrates analyses according to strict thresholds and panel B according to relaxed thresholds (for more methods and thresholds details see text and Table 1). The Scale of proportions for both panels is also shown.

For the strict set of thresholds the *median percent concordance* in non-clustered sequences was overall high (98%; IQR 94‒99%; range 81‒100%; Fig. 6A; Supplementary Table S7); RAxML had the highest *mean summary percent concordance* (97%), followed by four other model-based methods at 96%, IQ-Tree ultra-fast bootstrap at 95% and MEGA MCL at 94%; while HIV-TRACE was the lowest (92%). For the relaxed set of thresholds, *median percent concordance* was 95% (IQR 87‒98%; range 70‒100%; Fig. 6B; Supplementary Table S8), with a tied highest range for RAxML, FastTree regular bootstrap and IQ-Tree regular bootstrap (94% each); and the lowest for PhyML aLRT (88%). Overall, the range of *mean summary percent concordance* between analyzed methods in this analysis was 92‒97% for the strict set of thresholds, and 88‒94% for the relaxed thresholds.

Taken together, based on the concordance analyses, RaxML-based analytical approaches demonstrated the highest average estimates (91%) within the strict set of thresholds. Three (RaxML-, FastTree regular bootstrap- and IQ-Tree regular bootstrap-based) analytical approaches had the highest *mean summary percent concordance* (86% each) in the relaxed set of thresholds.

## Discussion

Real time identification of HIV clusters can and should trigger public health interventions to disrupt HIV transmissions and end the epidemic. In this study, we addressed whether analytical approaches differ in identification of HIV clusters, and examined concordance between most commonly used analytical approaches in identifying molecular HIV clusters. Since there is no gold standard for a ‘true’ HIV cluster we did not attempt to ‘validate’ clustering methods. Instead, we aimed to determine the extent of congruence and disagreement across approaches on the same empirical dataset. By comprehensively comparing cluster identification within and between commonly-used methods in a densely sampled dataset of 1886 HIV-1 subtype B *pol* sequences in Rhode Island, we demonstrate that the choice of methods and thresholds can lead to considerable variation in cluster characterization, which may impact the outcomes of integration of these data into public health activities.

Our key findings include, first, the demonstration that different analytical approaches can result in diverse identification of HIV-1 molecular clusters. These findings, supported by the within and between methods comparisons in identifying HIV molecular clusters, demonstrate that distance thresholds have more noticeable effects on clustering than topological support. The choice of distance threshold stringency may be more important for identifying molecular HIV clusters than the choice of software tool or analytic method. Second, the use of a range of criteria demonstrated heterogeneity in cluster identification among analytical approaches, which was more pronounced in stringent topological support and relaxed distance thresholds. Lastly, our introduction of the concordance analyses further demonstrated some differences across methods, supporting the same conclusion. This innovative analysis examines not only overall cluster proportions but also specific sequences in each cluster and is likely to have significant impact on public health actions that are triggered by cluster identification.

The demonstrated variation in cluster identification can impact public health outcomes. We suggest that proper selection of analytical approaches may need to be guided by a public health and/or scientific goal. Thus, strict thresholds may be more appropriate for targeting rapidly-growing clusters, disrupting transmission outbreaks or evaluating an HIV epidemic with low viral diversity^3,18–20^. In contrast, relaxed thresholds may be more appropriate for routine public health tracking of molecular HIV clusters to inform standard partner notifications and identify new HIV infections or cases not linked to care; or to perform an evolutionary analysis of an HIV-1 epidemic over longer time periods, to account for specifics of sampling and intra-host diversification of viral quasi-species over time. These recommendations, which address some of the outlined research gaps, will be informed by public health practitioners as these molecular epidemiology approaches are increasingly used in public health, and need to be scientifically investigated. Importantly, augmentation of phylogeny by epidemiologic data, not performed here but recently reported^14,21^, has the potential to further enhance understanding of HIV dynamics and public health guidance, and should be further explored.

Comparison of clustering outcomes between model-based methods and the distance-based HIV-TRACE is of particular interest because of the abundant recent use of the latter and its incorporation into Departments of Health activities for outbreak investigations^2^, at least in the USA. We confirmed previous reports^22^ that HIV-TRACE clustering outcomes are quite sensitive to the selection of the distance threshold. We additionally found that at compatible levels of distance thresholds, model-based methods identified less sequences in clusters. This should not be surprising, as node support, available in model-based methods but not HIV-TRACE, adds more stringency to clustering, though the distance estimation compatibility between distance- and model-based methods should be further examined. It is important to recognize these differences, as method and threshold choices could have implications for public health activities. Within the phylogenetic methods, node support values are not equal. For example, aLRT is a measurement of non-zero branch length, while traditional bootstrap is measurement of the proportion of sites that support a particular clade. It is likely that these differences contribute to the observed disagreement between the model-based methods analyzed. Notably, identified discordance among analytical approaches should not imply inferiority, and different tools, including development of new ones^23–29^, should be used for different scientific and epidemiological scenarios.

We note several limitations of the study. First, despite the densely sampled epidemic in Rhode Island (available sequences from ~ 80% of the state’s individuals with HIV), some sequencing data are still missing, which may alter clustering, and therefore our results. Second, we utilized partial HIV-1 *pol* sequences. Longer viral sequences may be more informative for identification of molecular HIV clusters^30–33^. Third, although we chose the most commonly-used analytical approaches for identification of clusters, there are other analytical approaches (e.g., Bayesian methods or alternative ways of measuring distance). Fourth, longitudinal evolution of clusters, which may be biologically relevant (i.e. clusters may change over time) and might impact discordance among analytical approaches, was not examined here. Lastly, we used a real-life dataset of only statewide HIV-1 sequences, which though beneficial and unique, mandates expansion with non-local sequences, as well as extension to other datasets, populations and geographic areas, to determine if and how sampling and epidemiological contexts influence the consistency of cluster identification by different analytical approaches.

In conclusion, this study brings new information on the diversity of HIV-1 cluster identification within and among commonly-used analytical approaches. Determination of thresholds stringency was found to be critical for HIV cluster identification. Among examined analytical approaches, all model-based methods except MEGA showed comparable performance and could be considered functionally equivalent using strict thresholds, with RaxML having the highest concordance with other methods. Using relaxed thresholds, RAxML, FastTree regular bootstrap and IQ-Tree regular bootstrap showed the highest concordance. Different methods, model- and distance-based, may be beneficial for diverse public health or scientific scenarios, supporting implementation and evaluation of HIV clustering-triggered public health activities. Recognizing the variability and limitations in cluster identification of commonly-used analytical approaches is an important step towards addressing the existing research gaps in developing HIV-clustering-triggered interventions to disrupt new HIV transmissions and end the HIV epidemic.

## Methods

### HIV-1 sequences

To compare clustering across methods, we used viral sequence data sampled from people living with HIV in Rhode Island, USA^34^. All HIV-1 sequences were generated through provider-ordered drug resistance testing, performed by certified commercial laboratories using the Sanger method. The vast majority of genotypes, particularly in later years, were obtained upon HIV diagnosis, as part of routine clinical care. The study was approved by, and a consent waiver was obtained from, the Institutional Review Board at The Miriam Hospital in Providence, RI. Sequence quality assessment and quality control and HIV-1 subtyping were performed with Stanford Database tools (https://hivdb.stanford.edu/). Overall, 3594 partial HIV-1 *pol* sequences (HXB2 nucleotide positions 2253–3554) from 2049 individuals sampled in Rhode Island during 2004–2018 were available. A total of 1,886 HIV-1 subtype B earliest (single per person) sequences were included in this study.

### Selection of methods and thresholds

To identify the most commonly used analytical approaches for HIV clustering, we conducted a PubMed (www.ncbi.nlm.nih.gov) search of English-written, recently published (2016–2019) papers, using search criteria keywords “HIV”, “transmission”, “cluster” and “clustering.” Of the 108 studies retrieved and reviewed (Supplementary Table S1), 31% used phylogenetic methods alone for cluster identification, 23% used distance-based methods alone, and 46% used a combination of methods. Supplementary Figure S1 summarizes the reviewed papers by publication year, targeted HIV-1 genes, sequencing methods, analyzed HIV-1 subtypes, excluding or including sites associated with HIV-1 drug-resistance, and usage of maximum likelihood (ML) models. The distributions of topological support thresholds and pairwise distances in the reviewed studies are presented in Supplementary Figures S2A and S2B, respectively.

Based on the review, we selected the following five software tools for model-based analyses, each of which infers phylogenies by ML: FastTree^35^ (used in 30% of studies), RAxML^36^ (29%), PhyML^37^ (23%), MEGA^38^ (18%), and IQ-Tree^39^. For FastTree we included both the Shimodaira-Hasegawa-like approximate likelihood ratio test (aLRT)^40^ and traditional bootstrap. For IQ-Tree we included both ultra-fast and traditional bootstrap. For MEGA^38^, we inferred phylogenies by maximum composite likelihood^41^ (MCL) and by the neighbor-joining (NJ) method with distances computed by the Tamura-Nei method^42^. In addition, for FastTree (traditional bootstrap), RAxML and IQ-Tree (both ultra-fast and traditional bootstrap), we included the alternative branch support calculated via transfer bootstrap expectation in BOOSTER^43^. The GTRCAT model was used to infer phylogenies in RAxML rapid bootstrap; the GTR model in FastTree, MEGA and PhyML; and the GTR + F + R10 model in IQ-Tree.

Overall, these 12 selected method combinations and variations represented the most commonly used analytical approaches for identification of molecular HIV clusters: (1) RAxML rapid bootstrap; (2) BOOSTER with RAxML rapid bootstrap replicates; (3) FastTree aLRT; (4) FastTree regular bootstrap; (5) BOOSTER with FastTree regular bootstrap replicates; (6) PhyML aLRT; (7) MEGA-MCL; (8) MEGA-NJ; (9) IQ-Tree ultra-fast bootstrap; (10) BOOSTER with IQ-Tree ultra-fast bootstrap replicates; (11) IQ-Tree regular bootstrap; and (12) BOOSTER with IQ-Tree regular bootstrap replicates. We also considered HIV-TRACE^22^, the most common distance-based method (used in 32% of the reviewed studies).

To examine the impact of specific thresholds in identification of molecular HIV-1 clusters, we used seven topological support cut-offs, from 0.70 to 1.0 in 0.05 increments (bootstrap or aLRT). We used 1000 bootstrap replicates for RAxML, FastTree, MEGA and IQ-Tree ultrafast. We used 100 replicates for IQ-Tree traditional bootstrap, due to the long runtime associated with this method.

To examine the impact of within- and between-method pairwise distance thresholds on cluster identification, we used seven thresholds: no distance threshold, 0.045, 0.030, 0.025, 0.020, 0.015, and 0.010 substitutions/site. Mean Tamura-Nei 93 (TN93)-corrected pairwise distances^42^ were estimated in R with pairwise deletion of gaps^44^. Comparisons of analytical approaches were performed across the 49 combinations of seven bootstrap/aLRT and seven distance thresholds that reflect the literature review (Supplementary Table S1). For HIV-TRACE, we used distance thresholds from 0.010 to 0.030 substitutions/site in 0.005 increments.

To identify molecular HIV clusters by model-based methods, sub-trees were extracted from inferred phylogenies and mean pairwise distances of each sub-tree were evaluated. Sub-trees that satisfied the pre-specified topological support and distance threshold were considered as clusters.

All methods were performed in accordance with relevant guidelines and regulations.

### Flow of cluster analysis, outcome parameters and statistical methods

The comparison of selected analytical approaches in identifying molecular HIV clusters was performed by (1) analysis of clustering outcomes *within* each method across a range of topological support and distance thresholds, (2) clustering comparison *between* methods, (3) analysis of differences between method-pairs in proportions of clustered sequences, and (4) concordance analysis of clustering including agreement between methods using several parameters (described below). The first two analyses (clustering *within* and *between* methods) included the 12 selected methods/variations. The clustering outcomes for these analyses included *proportions* of individuals in clusters and *number* of identified clusters. The next two analyses (differences in proportions and concordance) were narrowed down to seven methods by eliminating the four BOOSTER versions and MEGA NJ, to reduce number of comparisons. Differences of proportions were computed using two sets of thresholds, strict and relaxed (details provided below). Clustering results of model-based methods were compared with HIV-TRACE.

In the first three analyses (measures within and between methods and proportion differences) only *aggregated* outcomes are presented, such as proportion and number of clustered sequences. To examine whether each *actual* pair of sequences that cluster does so consistently across methods, we conducted a concordance analysis. Given the results from a pair of methods A and B, we assessed concordance using (1) proportion of pairs of sequences that cluster together by method A that also cluster together by method B (and vice versa; the proportion of pairs of sequences that cluster together by method B that also cluster together by method A); (2) proportion of clusters identified by method A that are identical for method B (consisting of exactly the same sequences) (and vice versa); and (3) proportion of sequences that are not in a cluster by method A that are also not in a cluster by method B (and vice versa*)*. Each concordance analysis was performed for all pairs of methods using strict and relaxed sets of thresholds (see below) and produced an asymmetric matrix presented as a heatmap. A *median percent concordance* (interquartile range (IQR) and range) was calculated for each matrix, summarizing each method-pair analysis. A *mean summary percent concordance* was calculated for each method and concordance measure, as the average of the concordance between the method and all other methods using the same threshold type (strict or relaxed, see below), including HIV-TRACE.

For the differences in proportions and concordance analyses we used two sets of thresholds, strict and relaxed, that were based on distinct combinations of topological support and distances (Table 1). The strict set of thresholds (topological support 0.95–1.0, distance 0.015) could be used when focusing on most recent transmission events, such as investigation of outbreaks or targeting rapidly-growing clusters and an explosive spread of HIV transmissions among high-risk populations. The relaxed set of thresholds (topological support 0.80–0.95, distance 0.030–0.045) could be used when focusing on more routine tracking of molecular HIV clusters to inform public health partner notifications, or when examining a historical evolution of local or global HIV epidemics with large numbers of transmissions over a long period of time. In such a scenario the extensive HIV intra-host viral evolution that accumulates over time and may result in lower topological support and larger distances than those used by strict thresholds, needs to be considered when identifying clusters. Thus, applying a strict rather than relaxed set of thresholds in this scenario would place fewer viral sequences into clusters and produce less informative outcomes.

## Supplementary information

Supplementary Information
