# Supplementary material for: Empirical comparison of analytical approaches for identifying molecular HIV-1 clusters
Source: Sci Rep. 2020 Oct 29;10:18547. doi: 10.1038/s41598-020-75560-1 (PMC7596705; doi:10.1038/s41598-020-75560-1)
Supplement: Supplementary file 1 — Supplementary Information [file 41598_2020_75560_MOESM1_ESM.pdf]

Supplementary Materials

Empirical Comparison of Analytical Approaches for Identifying Molecular HIV-1 Clusters

Vlad Novitsky[1§], Jon A. Steingrimsson[1], Mark Howison[2], Fizza S. Gillani[1], Yuanning Li[3], Akarsh Manne[1], John Fulton[1], Matthew Spence[4], Zoanne Parillo[4], Theodore Marak[4], Philip A Chan[1,4], Thomas Bertrand[4], Utpala Bandy[4], Nicole Alexander-Scott[4], Casey W. Dunn[3], Joseph Hogan[1], Rami Kantor[1§]

[1] Brown University, Providence, RI, USA
[2] Research Improving People's Life, Providence, RI, USA
[3] Yale University, New Haven, CT; USA
[4] Rhode Island Department of Health, Providence, RI, USA.

Supplementary Table S1. Characteristics of 108 reviewed papers using common analytical approaches for identifying HIV clusters.

Author	Year	Journal	Location	Sample size, individuals	Sampling years	HIV-1 gene	Distance method	Distance threshold, substitutions/site	Phylogeny	Method	Model	Bootstrap / SH-aLRT /Posterior Probability threshold
Araujo et. al.[1]	2019	Sci Rep	Portugal	65	2007-2017	PR + RT (partial)		0.003; 0.050	ML + Bayesian	PhyML + BEAST	GTR+G4+I	SH-aLRT ≥0.95 + PostProb ≥0.99
Arendt et. al.[2]	2019	PLoS One	Luxembourg	56	2013-2017	PR + RT (partial)			ML	FastTree	GTR+CAT	SH-aLRT ≥0.95
Billock et. al.[3]	2019	JAIDS	NC, USA	8,202	2010-2017	PR + RT (partial)	HIV-TRACE	0.015				
Chaillon et. al.[4]	2019	Open Forum Infect Dis	France; Quebec	1,862	1996-2016	PR + RT (partial)		0.010; 0.015				
Chung et. al.[5]	2019	PLoS One	Korea	927	1999-2012	PR + RT (partial)		0.015	NJ + ML	MEGA	GTR+G+I	Bootstrap ≥0.90
Crispim et. al.[6]	2019	Transfusion	Brazil	227	2011-2017	PR + RT (partial)			ML	PhyML		SH-aLRT ≥0.90
Dasgupta et. al.[7]	2019	ARHR	Michigan, Washington; USA	6,350	2008-2014	PR + RT (partial)	HIV-TRACE	0.015				
Dennis et. al.[8]	2019	JID	NC, USA	14,921	1997-2014	PR + RT (partial)		0.035	ML	FastTree		SH-aLRT ≥0.90
Fabeni et. al.[9]	2019	Sex Transm Infect	Italy	1,890	2005-2017	PR + RT (partial)	HIV-TRACE	0.015	ML + Bayesian	RAxML + MrBayes	GTR+G+I	Bootstrap ≥0.90 + PostProb ≥1.0
Gibson et. al.[10]	2019	Front Microbiol	DC, USA	1,996	1987-2015	PR + RT (partial)	HIV-TRACE	0.010	ML	RAxML		Bootstrap ≥0.70
Kostaki et. al.[11]	2019	Front Microbiol	Spain	391	2000-2014	PR + RT (partial)			ML + Bayesian	RAxML + MrBayes	GTR+G4	Bootstrap ≥0.75 + PostProb ≥0.82
Kusejko et. al.[12]	2019	JID	Switzerland	11,915	1988-2017	PR + RT (partial)		0.015 - 0.045	ML	FastTree	GTR+CAT	
Leung et. al.[13]	2019	Curr HIV Res	Hong Kong	2,315	1994-2013	PR + RT (partial)			ML	PhyML	GTR+G	Bootstrap ≥0.70
Nicolas et. al.[14]	2019	Clin Microbiol Infect	Spain	346	1997-2015	NA			NJ	NA	K2P	Bootstrap ≥0.90
Paraskevis et. al.[15]	2019	BMC Med	Spain, UK, Germany, Canada	8,955	by 2014	PR + RT (partial)			ML	RAxML	GTR+G	Bootstrap ≥0.75
Ragonnet-Cronin et. al.[16]	2019	Lancet HIV	LA, USA	22,398	2006-2016	PR + RT (partial)	HIV-TRACE	0.015				
Ratmann et. al.[17]	2019	Nat Commun	Uganda	2,652	2011-2015	NFLG	Phyloscanner	0.025				
Rhee et. al.[18]	2019	Open Forum Infect Dis	NC, USA	4,553	1998-2016	PR + RT (partial)	HIV-TRACE	0.015				
Stecher et. al.[19]	2019	Clin Microbiol Infect	Germany	714	2001-2016	PR + RT (partial)	HIV-TRACE	0.015				
Stecher et. al.[20]	2019	CID	Germany	714	2001-2016	PR + RT (partial)	HIV-TRACE	0.015				
Todesco et. al.[21]	2019	AIDS	France	70	2012-2013	PR + RT (partial)	HyPhy v2.1.2	0.015 - 0.045	ML	FastTree	GTR+G	SH-aLRT ≥0.70
Villandre et. al.[22]	2019	PloS One	Canada	3916	2002-2016	PR + RT (partial)	Gap Procedure	0.015 - 0.045	ML + Bayesian	RAxML BrBayes	GTR+G+I	Bootstrap ≥ 0.70;0.90;0.95%
Wu et. al.[23]	2019	BMC Infect Dis	China	362	2017-2018	PR + RT (partial)		0.015	ML	PhyML	GTR	SH-aLRT ≥0.90
Zuckerman et. al.[24]	2019	AIDS	Israel	502	2005-2016	PR + RT (partial)			Bayesian	BEAST	GTR+G+I	PostProb ≥0.90
Arimide et. al.[25]	2018	PLoS One	Ethiopia	301	2011-2013	PR + RT (partial)			ML	PhyML	GTR+G+I	SH-aLRT ≥0.90
Chang et. al.[26]	2018	J Int AIDS Soc	Thailand	288	2009-2015	*env*		0.030	ML	RAxML	GTR+G+I	Bootstrap ≥0.70
Chen et. al.[27]	2018	Virulence	China	117	2010-2013	*pol* and other genes	Cluster Picker	0.040	ML	MEGA	NA	NA
Chen et. al.[28]	2018	PLoS One	China	190	2013-2015	*pol* and other genes		0.030	NJ	MEGA	K2P	Bootstrap ≥0.90
Dalai et. al.[29]	2018	Front Microbiol	CA, USA	316	1996-2010	PR + RT (partial)	HIV-TRACE	0.020	ML	RAxML	GTR+G+I	Bootstrap ≥0.90
Dennis et. al.[30]	2018	Sex Transm Dis	NC, USA	68	2013-2014	PR + RT (partial)		0.015	ML	RAxML		Bootstrap ≥0.98
Dennis et. al.[31]	2018	ARHR	TN, USA	2915	2001-2015	PR + RT (partial)		0.015	ML	FastTree		

Author	Year	Journal	Location	Sample size, individuals	Sampling years	HIV-1 gene	Distance method	Distance threshold, substitutions/site	Phylogeny	Method	Model	Bootstrap / SH-aLRT /Posterior Probability threshold
Gonzalez-Domenech et. al.[32]	2018	PLoS One	Spain	57	2011-2016	PR + RT (partial)			ML + Bayesian	PhyML + MrBayes		Bootstrap 0.55-0.76 + PostProb 0.89-0.95
Hassan et. al.[33]	2018	PLoS One	Kenya	97	2005-2017	PR + RT (partial)		0.060	ML	PhyML		SH-aLRT ≥0.90
Kosakovsky Pond et. al.[34]	2018	Mol Biol Evol	USA	NA	NA	PR + RT (partial)	HIV-TRACE	0.015				
Kostaki et. al.[35]	2018	Curr HIV Res	Greece	104	NA	NFLG			ML	RAxML + FastTree		Bootstrap ≥0.70-0.90
Kostaki et. al.[36]	2018	ARHR	Russia, Central Asia	142	2001-2013	PR + RT (partial)			ML	RAxML	GTR+CAT	
Kostaki et. al.[37]	2018	JID	Greece	119	2013-2015	PR + RT (partial)			ML	RAxML + FastTree + MrBayes		Bootstrap ≥0.70-0.90 + PostProb ≥0.80
Kroon et. al.[38]	2018	AIDS	Thailand	439	2009-2017	PR + RT (partial)		0.015	ML	MEGA	K2P	Bootstrap ≥0.80
Levintow et. al.[39]	2018	Open Forum Infect Dis	NC, USA	15,246	1997-2014	PR + RT (partial)		0.035	ML	FastTree	GTR	SH-aLRT ≥0.90
Lopez et. al.[40]	2018	ARHR	Puerto Rico	846	2013-2017	PR + RT (partial)			ML	MEGA		Bootstrap ≥0.70
Lu et. al.[41]	2018	ARHR	China	254	2013	*pol* and other genes			ML	MEGA		Bootstrap ≥0.75
Mbisa et. al.[42]	2018	CID	UK	835	2011-2015	PR + RT (partial)		0.015; 0.045	ML	FastTree	GTR	SH-aLRT ≥0.90
Morgan et. al.[43]	2018	AJPH	Chicago, USA	618	2013 - 2016	PR + RT (partial)		0.015	NJ	MEGA	TN93	
Oster et. al.[44]	2018	JAIDS	USA	156,553	2013-2015	PR + RT (partial)	HIV-TRACE	0.005; 0.015				
Pasquale et. al.[45]	2018	JAIDS	NC, USA	15,246	1997-2014	PR + RT (partial)		0.035	ML + Bayesian	FastTree	GTR	SH-aLRT ≥0.90 + PostProb ≥0.95
Patino-Galindo et. al.[46]	2018	Infect Genet Evol	Spain	23	1989-2014	NFLG			ML + Bayesian	PhyML	GTR	
Pineda-Pena et. al.[47]	2018	Sci Rep	Cyprus	336	1986-2012	PR + RT (partial)		0.045	ML	RAxML	GTR+G4	Bootstrap ≥0.70
Ragonnet-Cronin et. al.[48]	2018	Lancet HIV	UK	63,065	2001-2013	PR + RT (partial)		0.045	ML	RAxML		Bootstrap ≥0.90
Ragonnet-Cronin et. al.[49]	2018	JID	Scotland, UK	2,572	2005 - 2016	PR + RT (partial)		0.010				
Sivay et. al.[50]	2018	PLoS One	South Africa	200	2011–2015	PR + RT (partial)	HIV-TRACE	0.025	ML	RAxML + FastTree	GTR+CAT	Bootstrap ≥0.90
Soto-Nava et. al.[51]	2018	J Virol	Mexico	1,612	2000-2014	*pol* and other genes		0.015	ML	FastTree	GTR	Bootstrap ≥0.90
Stecher et. al.[52]	2018	Sci Rep	Germany	2,774	1999-2016	PR + RT (partial)	HIV-TRACE	0.015				
Verhofstede et. al.[53]	2018	Infect Genet Evol	Belgium	1,665	2013-2015	PR + RT (partial)		0.015	ML	PhyML	GTR+G+I	SH-aLRT ≥0.97
Volz et. al.[54]	2018	JID	UK	6,912	1991-2014	PR + RT (partial)		0.005; 0.015	ML	RAxML	GTR+G	
Wang et. al.[55]	2018	PLoS One	China	542	2014-2015	PR + RT (partial)		0.015	ML	FastTree	GTR+G	Bootstrap ≥0.70
Wertheim et. al.[56]	2018	JID	NY, USA	65,736	2005-2017	PR + RT (partial)	HIV-TRACE	0.015				
Yang et. al.[57]	2018	Medicine (Baltimore)	China	323	2012-2015	PR + RT (partial)			NJ + ML	FastTree + MEGA	K2P (nj)	SH-aLRT ≥0.90
Yebra et. al.[58]	2018	PLoS One	UK	420	2012-2014	NFLG		0.060	ML	RAxML		Bootstrap ≥0.90
Ahn et. al.[59]	2017	ARHR	South Korea	143	2013-2014	*env*	HIV-TRACE	0.020				
Brand et. al.[60]	2017	AIDS	France	549	2012-2014	*env*	MEGA K2P	0.015				
Brenner et. al.[61]	2017	AIDS	Canada	3,901	2002-2015	PR + RT (partial)		0.015	NJ	MEGA		Bootstrap ≥0.95
Brenner et. al.[62]	2017	J Antimicrob Chemother	Canada	4,039	2002-2015	*pol* and other genes		0.015	NJ	MEGA		Bootstrap ≥0.95
Chaillon et. al.[63]	2017	Infect Genet Evol	South America	6,092	2011-2016	PR + RT (partial)	HIV-TRACE	0.015				
Chaillon et. al.[64]	2017	Retrovirology	France	1,356	1999-2014	PR + RT (partial)		0.015				

Author	Year	Journal	Location	Sample size, individuals	Sampling years	HIV-1 gene	Distance method	Distance threshold, substitutions/site	Phylogeny	Method	Model	Bootstrap / SH-aLRT /Posterior Probability threshold
Dennis et. al.[65]	2017	Virus Evol	NC, USA	325	1997-2014	PR + RT (partial)		0.035	ML	FastTree		SH-aLRT ≥0.90
Fabeni et. al.[66]	2017	J Antimicrob Chemother	Italy	4,323	2000-2014	PR + RT (partial)		0.015	ML + Bayesian	RAxML + MrBayes	GTR+G+I	Bootstrap ≥0.90 + PostProb ≥1.0
Fearnhill et. al.[67]	2017	CID	Ukraine	876	2013-2015	PR + RT (partial)		0.025	ML	FastTree	GTR	SH-aLRT ≥0.80
Hakre et. al.[68]	2017	PLoS One	USA	518	2001-2012	PR + RT (partial)		0.015	ML	MEGA	K2P	Bootstrap ≥0.95
Jagdagsuren et. al.[69]	2017	PLoS One	Mongolia	143	2010-2016	*pol* and other genes			NJ + Bayesian	MEGA + BEAST	K2P	Bootstrap ≥0.90 + PostProb ≥0.90
Morgan et. al.[70]	2017	JAIDS	Chicago, USA	86	2013-2016	PR + RT (partial)		0.015	NJ			
Mozhgani et. al.[71]	2017	Intervirology	Iran	50	NA	*gag* and *env*			ML	MEGA	K2P	Bootstrap ≥0.80
Neogi et. al.[72]	2017	Sci Rep	Sweden	5,246	1993 - 2016	NFLG	RAxML, GTR+G	0.020; 0.080				
Paraschiv et. al.[73]	2017	PLoS One	Romania	117	2011-2014	PR + RT (partial)			ML + Bayesian	FastTree + BEAST	GTR+G+I	SH-aLRT ≥0.90 + PostProb ≥1.0
Parczewski et. al.[74]	2017	PLoS One	Poland	966	1989-2014	PR + RT (partial)		0.030	ML	PhyML	GTR+G	SH-aLRT ≥0.90
Patino-Galindo et. al.[75]	2017	Sci Rep	Spain	1,804	2004-2014	PR + RT (partial)			ML	FastTree + PhyML		SH-aLRT ≥0.98
Perez-Losada et. al.[76]	2017	PLoS One	DC, USA	1,882	2011-2015	*pol* and other genes	HIV-TRACE	0.010	ML	RAxML + MrBayes		Bootstrap ≥0.70 + PostProb ≥0.95
Ratmann et. al.[77]	2017	ARHR	Uganda, Botswana, South Africa	3,985	2009-2014	NFLG			ML	RAxML + FastTree + PhyML + IQ-Tree		
Rose et. al.[78]	2017	ARHR	Uganda	1,054	2008-2009	*env*	HIV-TRACE	0.053	ML	PhyML	HKY+G4; GTR+G4	
Sallam et. al.[79]	2017	Infect Genet Evol	Iceland	230	1985-2012	PR + RT (partial)			ML	GARLI		SH-aLRT ≥0.90
Sallam et. al.[80]	2017	Heliyon	Middle East; North America	2,036	1988-2016	PR + RT (partial)			ML	PhyML + GARLI	GTR+G+I	SH-aLRT ≥0.90
Temereanca et. al.[81]	2017	Rom Biotechnol Lett	Romania	37	2010-2013	PR + RT (partial)	HIV-TRACE	0.015	ML	PhyML	TN93	
Valverde et. al.[82]	2017	JAIDS	USA	12,064	2001-2013	PR + RT (partial)	HIV-TRACE	0.015				
Vanhommerig et. al.[83]	2017	AIDS	Netherlands	5,038	1998-2014	PR + RT (partial)		0.080	ML	FastTree	GTR+G	SH-aLRT ≥0.90
Vrancken et. al.[84]	2017	Infect Genet Evol	Canada	1,146	2007-2013	PR + RT (partial)			Bayesian	BEAST	HKY+G	PostProb ≥0.95
Wang et. al.[85]	2017	Sci Rep	China	2,965	NA	PR + RT (partial)			ML	PhyML	GTR+G+I	SH-aLRT ≥0.90
Wertheim et. al.[86]	2017	PLoS Pathog	NY, USA	1,342	2006-2012	PR + RT (partial)	HIV-TRACE	0.0025; 0.040				
Wolf et. al.[87]	2017	ARHR	Seattle, USA	1,953	2000-2013	PR + RT (partial)		0.015	ML	FastTree	GTR+G	SH-aLRT ≥0.95
Castley et. al.[88]	2016	ARHR	Australia	1,021	2000-2014	PR + RT (partial)		0.015	ML	MEGA		Bootstrap ≥0.98
Chin et. al.[89]	2016	J Med Virol	South Korea	131	2013-2014	PR + RT (partial)		0.015				
Esbjornsson et. al.[90]	2016	Virus Evol	Sweden, Denmark, and Finland	3,202	2000-2012	PR + RT (partial)			ML	PhyML + GARLI		SH-aLRT ≥0.90
Hoenigl et. al.[91]	2016	PLoS One	Australia	259	2008-2014	PR + RT (partial)		0.015	ML	RAxML		Bootstrap ≥0.80
Junqueira et. al.[92]	2016	PLoS One	South America	4,810	1989-2013	PR + RT (partial)		0.010 - 0.075	ML	RAxML	GTR+G+I	SH-aLRT ≥0.90
Konou et. al.[93]	2016	Infect Genet Evol	Togo	75	2011-2013	PR + RT (partial)			ML	PhyML		Bootstrap ≥0.98
Magiorkinis et. al.[94]	2016	Infect Genet Evol	Europe, North America	6,688	1996-2007	PR + RT (partial)			ML	RAxML	GTR+G	
Marzel et. al.[95]	2016	CID	Switzerland	10,970	1984-2014	PR + RT (partial)		0.010 - 0.025	ML	FastTree	GTR	Bootstrap ≥0.50-1.0
Mehta et. al.[96]	2016	CID	San Diego, USA	652	1996–2012	PR + RT (partial)		0.015				

Author	Year	Journal	Location	Sample size, individuals	Sampling years	HIV-1 gene	Distance method	Distance threshold, substitutions/site	Phylogeny	Method	Model	Bootstrap / SH-aLRT /Posterior Probability threshold
Panichsillapakit et. al.[97]	2016	JAIDS	San Diego, USA	496	1996-2013	PR + RT (partial)			ML	FastTree	GTR+G	Bootstrap ≥0.70
Patino-Galindo et. al.[98]	2016	Infect Genet Evol	Spain	2,497	2001-2008	PR + RT (partial)			ML	FastTree		SH-aLRT ≥0.90-0.999
Pines et. al.[99]	2016	AIDS	San Diego, USA	986	1996-2015	PR + RT (partial)	HIV-TRACE	0.015				
Poon et. al.[100]	2016	Lancet HIV	British Columbia, Canada	8,800	1999-2015	PR + RT (partial)	in-house method	0.020				
Pouran Yousef et. al.[101]	2016	JAIDS	Germany	1,159	1997-2012	PR + RT (partial)		0.013	ML	RAxML	GTR+CAT	Bootstrap ≥0.95
Ragonnet-Cronin et. al.[102]	2016	JID	UK	15,000	2007-2009	PR + RT (partial)		0.045	ML	RAxML		Bootstrap ≥0.90
Ragonnet-Cronin et. al.[103]	2016	Sci Rep	UK and Switzerland	9,956	1995-2014	PR + RT (partial)		0.015; 0.045	ML	FastTree		Bootstrap ≥0.70-0.80-0.90-0.95
Shao et. al.[104]	2016	J Med Virol	China	81	2013-2014	*gag*			ML	PhyML		
Viciana et. al.[105]	2016	J Antimicrob Chemother	Spain	30	2007 - 2014	PR + RT (partial)			NJ	Phylip	K2P	
Wertheim et. al.[106]	2016	ARHR	USA	127,754	2001-2012	PR + RT (partial)	HIV-TRACE	0.015	Bayesian	BEAST		PostProb ≥0.95
Yebra et. al.[107]	2016	Sci Rep	NA	4,662	1989-2011	*pol* and other genes			ML	RAxML	GTR+G	
Zeh et. al.[108]	2016	PLoS One	Kenya	258	2003-2005	PR + RT (partial)		0.015	NJ + ML	MEGA		Bootstrap ≥0.85

Supplementary References (reviewed papers, n=108):

1 Araujo, P. M. M., Carvalho, A., Pingarilho, M., Abecasis, A. B. & Osorio, N. S. Characterization of a large cluster of HIV-1 A1 infections detected in Portugal and connected to several Western European countries. *Scientific reports* **9**, 7223, doi:10.1038/s41598-019-43420-2 (2019).

2 Arendt, V. *et al.* Injection of cocaine is associated with a recent HIV outbreak in people who inject drugs in Luxembourg. *PLoS One* **14**, e0215570, doi:10.1371/journal.pone.0215570 (2019).

3 Billock, R. M. *et al.* Prediction of HIV Transmission Cluster Growth With Statewide Surveillance Data. *J Acquir Immune Defic Syndr* **80**, 152-159, doi:10.1097/QAI.0000000000001905 (2019).

4 Chaillon, A. *et al.* In-depth Sampling of High-risk Populations to Characterize HIV Transmission Epidemics Among Young MSM Using PrEP in France and Quebec. *Open forum infectious diseases* **6**, ofz080, doi:10.1093/ofid/ofz080 (2019).

5 Chung, Y. S. *et al.* Phylogenetic transmission clusters among newly diagnosed antiretroviral drug-naive patients with human immunodeficiency virus-1 in Korea: A study from 1999 to 2012. *PLoS One* **14**, e0217817, doi:10.1371/journal.pone.0217817 (2019).

6 Esashika Crispim, M. A., da Guarda Reis, M. N., Fraiji, N., Bello, G. & Stefani, M. M. A. Detection of human immunodeficiency virus Type 1 phylogenetic clusters with multidrug resistance mutations among 2011 to 2017 blood donors from the highly endemic Northern Brazilian Amazon. *Transfusion*, doi:10.1111/trf.15347 (2019).

7 Dasgupta, S. *et al.* Estimating Effects of HIV Sequencing Data Completeness on Transmission Network Patterns and Detection of Growing HIV Transmission Clusters. *AIDS Res Hum Retroviruses* **35**, 368-375, doi:10.1089/AID.2018.0181 (2019).

8 Dennis, A. M. *et al.* HIV-1 Phylodynamics to Detect and Characterize Active Transmission Clusters in North Carolina. *J Infect Dis*, doi:10.1093/infdis/jiz176 (2019).

9 Fabeni, L. *et al.* Characterisation of HIV-1 molecular transmission clusters among newly diagnosed individuals infected with non-B subtypes in Italy. *Sex Transm Infect*, doi:10.1136/sextrans-2019-054017 (2019).

10 Gibson, K. M. *et al.* A 28-Year History of HIV-1 Drug Resistance and Transmission in Washington, DC. *Frontiers in microbiology* **10**, 369, doi:10.3389/fmicb.2019.00369 (2019).

11 Kostaki, E. G. *et al.* Spatiotemporal Characteristics of the Largest HIV-1 CRF02_AG Outbreak in Spain: Evidence for Onward Transmissions. *Frontiers in microbiology* **10**, 370, doi:10.3389/fmicb.2019.00370 (2019).

12 Kusejko, K. *et al.* A Systematic Phylogenetic Approach to Study the Interaction of HIV-1 With Coinfections, Noncommunicable Diseases, and Opportunistic Diseases. *J Infect Dis* **220**, 244-253, doi:10.1093/infdis/jiz093 (2019).

13 Leung, K. S. *et al.* Molecular characterization of HIV-1 minority subtypes in Hong Kong: A recent epidemic of CRF07_BC among the men who have sex with men population. *Current HIV research*, doi:10.2174/1570162x17666190530081355 (2019).

14 Nicolas, D. *et al.* Epidemiological changes of acute/recent human immunodeficiency virus type 1 infection in Barcelona, Spain (1997-2015): a prospective cohort study. *Clin Microbiol Infect* **25**, 878-884, doi:10.1016/j.cmi.2018.10.021 (2019).

15 Paraskevis, D. *et al.* HIV-1 molecular transmission clusters in nine European countries and Canada: association with demographic and clinical factors. *BMC medicine* **17**, 4, doi:10.1186/s12916-018-1241-1 (2019).

16 Ragonnet-Cronin, M. *et al.* HIV transmission networks among transgender women in Los Angeles County, CA, USA: a phylogenetic analysis of surveillance data. *The lancet. HIV* **6**, e164-e172, doi:10.1016/s2352-3018(18)30359-x (2019).

17 Ratmann, O. *et al.* Inferring HIV-1 transmission networks and sources of epidemic spread in Africa with deep-sequence phylogenetic analysis. *Nature communications* **10**, 1411, doi:10.1038/s41467-019-09139-4 (2019).

18 Rhee, S. Y. *et al.* National and International Dimensions of Human Immunodeficiency Virus-1 Sequence Clusters in a Northern California Clinical Cohort. *Open forum infectious diseases* **6**, ofz135, doi:10.1093/ofid/ofz135 (2019).

19 Stecher, M. *et al.* Pretreatment human immunodeficiency virus type 1 (HIV-1) drug resistance in transmission clusters of the Cologne-Bonn region, Germany. *Clin Microbiol Infect* **25**, 253.e251-253.e254, doi:10.1016/j.cmi.2018.09.025 (2019).

20 Stecher, M. *et al.* Hotspots of Transmission Driving the Local Human Immunodeficiency Virus Epidemic in the Cologne-Bonn Region, Germany. *Clin Infect Dis* **68**, 1539-1546, doi:10.1093/cid/ciy744 (2019).

21 Todesco, E. *et al.* Caution is needed in interpreting HIV transmission chains by ultradeep sequencing. *Aids* **33**, 691-699, doi:10.1097/qad.0000000000002105 (2019).
22 Villandre, L. *et al.* Assessing the role of transmission chains in the spread of HIV-1 among men who have sex with men in Quebec, Canada. *PLoS One* **14**, e0213366, doi:10.1371/journal.pone.0213366 (2019).
23 Wu, J. *et al.* Phylogenetic analysis highlights the role of older people in the transmission of HIV-1 in Fuyang, Anhui Province, China. *BMC Infect Dis* **19**, 562, doi:10.1186/s12879-019-4187-9 (2019).
24 Zuckerman, N. S. *et al.* Sexual intermingling of Arab and Jewish MSM in Israel: results of a molecular epidemiology study. *AIDS* **33**, 339-344, doi:10.1097/QAD.0000000000002057 (2019).
25 Arimide, D. A. *et al.* HIV-genetic diversity and drug resistance transmission clusters in Gondar, Northern Ethiopia, 2003-2013. *PLoS One* **13**, e0205446, doi:10.1371/journal.pone.0205446 (2018).
26 Chang, D. *et al.* Molecular epidemiology of a primarily MSM acute HIV-1 cohort in Bangkok, Thailand and connections within networks of transmission in Asia. *J Int AIDS Soc* **21**, e25204, doi:10.1002/jia2.25204 (2018).
27 Chen, X. *et al.* Burmese injecting drug users in Yunnan play a pivotal role in the cross-border transmission of HIV-1 in the China-Myanmar border region. *Virulence* **9**, 1195-1204, doi:10.1080/21505594.2018.1496777 (2018).
28 Chen, M. *et al.* HIV-1 genetic transmission networks among men who have sex with men in Kunming, China. *PLoS One* **13**, e0196548, doi:10.1371/journal.pone.0196548 (2018).
29 Dalai, S. C. *et al.* Combining Phylogenetic and Network Approaches to Identify HIV-1 Transmission Links in San Mateo County, California. *Frontiers in microbiology* **9**, 2799, doi:10.3389/fmicb.2018.02799 (2018).
30 Dennis, A. M. *et al.* Integration of Contact Tracing and Phylogenetics in an Investigation of Acute HIV Infection. *Sex Transm Dis* **45**, 222-228, doi:10.1097/olq.0000000000000726 (2018).
31 Dennis, A. M. *et al.* HIV-1 Transmission Clustering and Phylodynamics Highlight the Important Role of Young Men Who Have Sex with Men. *AIDS Res Hum Retroviruses* **34**, 879-888, doi:10.1089/aid.2018.0039 (2018).
32 Gonzalez-Domenech, C. M. *et al.* Emergence as an outbreak of the HIV-1 CRF19_cpx variant in treatment-naive patients in southern Spain. *PLoS One* **13**, e0190544, doi:10.1371/journal.pone.0190544 (2018).
33 Hassan, A. S. *et al.* HIV-1 subtype diversity, transmission networks and transmitted drug resistance amongst acute and early infected MSM populations from Coastal Kenya. *PLoS One* **13**, e0206177, doi:10.1371/journal.pone.0206177 (2018).
34 Kosakovsky Pond, S. L., Weaver, S., Leigh Brown, A. J. & Wertheim, J. O. HIV-TRACE (TRAnsmission Cluster Engine): a Tool for Large Scale Molecular Epidemiology of HIV-1 and Other Rapidly Evolving Pathogens. *Mol Biol Evol* **35**, 1812-1819, doi:10.1093/molbev/msy016 (2018).
35 Kostaki, E. G. *et al.* Near Full-length Genomic Sequencing and Molecular Analysis of HIV-Infected Individuals in a Network-based Intervention (TRIP) in Athens, Greece: Evidence that Transmissions Occur More Frequently from those with High HIV-RNA. *Curr HIV Res* **16**, 345-353, doi:10.2174/1570162x17666190130120757 (2018).
36 Kostaki, E. G. *et al.* Spatiotemporal Characteristics of the HIV-1 CRF02_AG/CRF63_02A1 Epidemic in Russia and Central Asia. *AIDS Res Hum Retroviruses* **34**, 415-420, doi:10.1089/aid.2017.0233 (2018).
37 Kostaki, E. G. *et al.* Molecular Analysis of Human Immunodeficiency Virus Type 1 (HIV-1)-Infected Individuals in a Network-Based Intervention (Transmission Reduction Intervention Project): Phylogenetics Identify HIV-1-Infected Individuals With Social Links. *J Infect Dis* **218**, 707-715, doi:10.1093/infdis/jiy239 (2018).
38 Kroon, E. *et al.* Transmission dynamics among participants initiating antiretroviral therapy upon diagnosis of early acute HIV-1 infection in Thailand. *Aids* **32**, 2373-2381, doi:10.1097/qad.0000000000001956 (2018).
39 Levintow, S. N. *et al.* Prevalence and Transmission Dynamics of HIV-1 Transmitted Drug Resistance in a Southeastern Cohort. *Open forum infectious diseases* **5**, ofy178, doi:10.1093/ofid/ofy178 (2018).
40 Lopez, P. *et al.* Molecular Epidemiology of HIV-1 Virus in Puerto Rico: Novel Cases of HIV-1 Subtype C, D, and CRF-24BG. *AIDS Res Hum Retroviruses* **34**, 507-516, doi:10.1089/AID.2017.0305 (2018).
41 Lu, X. *et al.* Regional Transmission Pattern of HIV-1 Non-CRF01_AE Strains Circulating in Hebei Province, China. *AIDS Res Hum Retroviruses* **34**, 222-227, doi:10.1089/aid.2017.0166 (2018).
42 Mbisa, J. L. *et al.* Determining the origins of HIV-1 drug-resistant minority variants in people who are recently infected using phylogenetic reconstruction. *Clin Infect Dis*, doi:10.1093/cid/ciy1048 (2018).
43 Morgan, E., Skaathun, B. & Schneider, J. A. Sexual, Social, and Genetic Network Overlap: A Socio-Molecular Approach Toward Public Health Intervention of HIV. *Am J Public Health* **108**, 1528-1534, doi:10.2105/AJPH.2018.304438 (2018).
44 Oster, A. M. *et al.* Identifying Clusters of Recent and Rapid HIV Transmission Through Analysis of Molecular Surveillance Data. *J Acquir Immune Defic Syndr* **79**, 543-550, doi:10.1097/QAI.0000000000001856 (2018).

45 Pasquale, D. K. *et al.* Leveraging Phylogenetics to Understand HIV Transmission and Partner Notification Networks. *J Acquir Immune Defic Syndr* **78**, 367-375, doi:10.1097/qai.0000000000001695 (2018).
46 Patino-Galindo, J. A. *et al.* Genome-scale analysis of evolutionary rate and selection in a fast-expanding Spanish cluster of HIV-1 subtype F1. *Infect Genet Evol* **66**, 43-47, doi:10.1016/j.meegid.2018.09.008 (2018).
47 Pineda-Pena, A. C. *et al.* HIV-1 Infection in Cyprus, the Eastern Mediterranean European Frontier: A Densely Sampled Transmission Dynamics Analysis from 1986 to 2012. *Scientific reports* **8**, 1702, doi:10.1038/s41598-017-19080-5 (2018).
48 Ragonnet-Cronin, M. *et al.* Non-disclosed men who have sex with men in UK HIV transmission networks: phylogenetic analysis of surveillance data. *The lancet. HIV* **5**, e309-e316, doi:10.1016/s2352-3018(18)30062-6 (2018).
49 Ragonnet-Cronin, M. *et al.* Recent and Rapid Transmission of HIV Among People Who Inject Drugs in Scotland Revealed Through Phylogenetic Analysis. *J Infect Dis* **217**, 1875-1882, doi:10.1093/infdis/jiy130 (2018).
50 Sivay, M. V. *et al.* HIV-1 diversity among young women in rural South Africa: HPTN 068. *PLoS One* **13**, e0198999, doi:10.1371/journal.pone.0198999 (2018).
51 Soto-Nava, M. *et al.* Weaker HLA Footprints on HIV in the Unique and Highly Genetically Admixed Host Population of Mexico. *J Virol* **92**, doi:10.1128/JVI.01128-17 (2018).
52 Stecher, M. *et al.* Molecular Epidemiology of the HIV Epidemic in Three German Metropolitan Regions - Cologne/Bonn, Munich and Hannover, 1999-2016. *Scientific reports* **8**, 6799, doi:10.1038/s41598-018-25004-8 (2018).
53 Verhofstede, C. *et al.* Phylogenetic analysis of the Belgian HIV-1 epidemic reveals that local transmission is almost exclusively driven by men having sex with men despite presence of large African migrant communities. *Infect Genet Evol* **61**, 36-44, doi:10.1016/j.meegid.2018.03.002 (2018).
54 Volz, E. M. *et al.* Molecular Epidemiology of HIV-1 Subtype B Reveals Heterogeneous Transmission Risk: Implications for Intervention and Control. *J Infect Dis* **217**, 1522-1529, doi:10.1093/infdis/jiy044 (2018).
55 Wang, X. *et al.* Epidemiological surveillance of HIV-1 transmitted drug resistance among newly diagnosed individuals in Shijiazhuang, northern China, 2014-2015. *PLoS One* **13**, e0198005, doi:10.1371/journal.pone.0198005 (2018).
56 Wertheim, J. O. *et al.* Growth of HIV-1 Molecular Transmission Clusters in New York City. *J Infect Dis* **218**, 1943-1953, doi:10.1093/infdis/jiy431 (2018).
57 Yang, Y. *et al.* Phylogenetic and temporal dynamics of human immunodeficiency virus type 1 CRF01_AE and CRF07_BC among recently infected antiretroviral therapy-naive men who have sex with men in Jiangsu province, China, 2012 to 2015: A molecular epidemiology-based study. *Medicine* **97**, e9826, doi:10.1097/md.0000000000009826 (2018).
58 Yebra, G. *et al.* A high HIV-1 strain variability in London, UK, revealed by full-genome analysis: Results from the ICONIC project. *PLoS One* **13**, e0192081, doi:10.1371/journal.pone.0192081 (2018).
59 Ahn, M. Y. *et al.* Short Communication: HIV-1 Transmission Networks Across South Korea. *AIDS Res Hum Retroviruses* **33**, 827-831, doi:10.1089/aid.2016.0212 (2017).
60 Brand, D. *et al.* HIV surveillance combining an assay for identification of very recent infection and phylogenetic analyses on dried spots. *AIDS* **31**, 407-416, doi:10.1097/QAD.0000000000001325 (2017).
61 Brenner, B. G. *et al.* Large cluster outbreaks sustain the HIV epidemic among MSM in Quebec. *Aids* **31**, 707-717, doi:10.1097/qad.0000000000001383 (2017).
62 Brenner, B. G. *et al.* HIV-1 strains belonging to large phylogenetic clusters show accelerated escape from integrase inhibitors in cell culture compared with viral isolates from singleton/small clusters. *J Antimicrob Chemother* **72**, 2171-2183, doi:10.1093/jac/dkx118 (2017).
63 Chaillon, A. *et al.* Identification of major routes of HIV transmission throughout Mesoamerica. *Infect Genet Evol* **54**, 98-107, doi:10.1016/j.meegid.2017.06.021 (2017).
64 Chaillon, A. *et al.* Spatiotemporal dynamics of HIV-1 transmission in France (1999-2014) and impact of targeted prevention strategies. *Retrovirology* **14**, 15, doi:10.1186/s12977-017-0339-4 (2017).
65 Dennis, A. M. *et al.* Rising prevalence of non-B HIV-1 subtypes in North Carolina and evidence for local onward transmission. *Virus Evol* **3**, vex013, doi:10.1093/ve/vex013 (2017).
66 Fabeni, L. *et al.* Dynamics and phylogenetic relationships of HIV-1 transmitted drug resistance according to subtype in Italy over the years 2000-14. *J Antimicrob Chemother* **72**, 2837-2845, doi:10.1093/jac/dkx231 (2017).

67 Fearnhill, E. *et al.* A Phylogenetic Analysis of Human Immunodeficiency Virus Type 1 Sequences in Kiev: Findings Among Key Populations. *Clin Infect Dis* **65**, 1127-1135, doi:10.1093/cid/cix499 (2017).

68 Hakre, S. *et al.* Characteristics of HIV-infected U.S. Army soldiers linked in molecular transmission clusters, 2001-2012. *PLoS One* **12**, e0182376, doi:10.1371/journal.pone.0182376 (2017).

69 Jagdagsuren, D. *et al.* The second molecular epidemiological study of HIV infection in Mongolia between 2010 and 2016. *PLoS One* **12**, e0189605, doi:10.1371/journal.pone.0189605 (2017).

70 Morgan, E., Nyaku, A. N., D'Aquila, R. T. & Schneider, J. A. Determinants of HIV Phylogenetic Clustering in Chicago Among Young Black Men Who Have Sex With Men From the uConnect Cohort. *J Acquir Immune Defic Syndr* **75**, 265-270, doi:10.1097/qai.0000000000001379 (2017).

71 Mozhgani, S. H. *et al.* CRF35-AD as the Main Circulating Genotype of Human Immunodeficiency Virus Type 1 Infection in Iran: A Phylogenetic and Demographic-Based Study. *Intervirology* **60**, 144-148, doi:10.1159/000484691 (2017).

72 Neogi, U. *et al.* Recent increased identification and transmission of HIV-1 unique recombinant forms in Sweden. *Scientific reports* **7**, 6371, doi:10.1038/s41598-017-06860-2 (2017).

73 Paraschiv, S. *et al.* Epidemic dispersion of HIV and HCV in a population of co-infected Romanian injecting drug users. *PLoS One* **12**, e0185866, doi:10.1371/journal.pone.0185866 (2017).

74 Parczewski, M. *et al.* Expanding HIV-1 subtype B transmission networks among men who have sex with men in Poland. *PLoS One* **12**, e0172473, doi:10.1371/journal.pone.0172473 (2017).

75 Patino-Galindo, J. A. *et al.* The molecular epidemiology of HIV-1 in the Comunidad Valenciana (Spain): analysis of transmission clusters. *Scientific reports* **7**, 11584, doi:10.1038/s41598-017-10286-1 (2017).

76 Perez-Losada, M. *et al.* Characterization of HIV diversity, phylodynamics and drug resistance in Washington, DC. *PLoS One* **12**, e0185644, doi:10.1371/journal.pone.0185644 (2017).

77 Ratmann, O. *et al.* HIV-1 full-genome phylogenetics of generalized epidemics in sub-Saharan Africa: impact of missing nucleotide characters in next-generation sequences. *AIDS Res Hum Retroviruses*, doi:10.1089/AID.2017.0061 (2017).

78 Rose, R. *et al.* Identifying Transmission Clusters with Cluster Picker and HIV-TRACE. *AIDS Res Hum Retroviruses* **33**, 211-218, doi:10.1089/AID.2016.0205 (2017).

79 Sallam, M. *et al.* Molecular epidemiology of HIV-1 in Iceland: Early introductions, transmission dynamics and recent outbreaks among injection drug users. *Infect Genet Evol* **49**, 157-163, doi:10.1016/j.meegid.2017.01.004 (2017).

80 Sallam, M. *et al.* Genetic characterization of human immunodeficiency virus type 1 transmission in the Middle East and North Africa. *Heliyon* **3**, e00352, doi:10.1016/j.heliyon.2017.e00352 (2017).

81 Temereanca, A. *et al.* HIV transmission clusters among injecting drug users in Romania. *Rom Biotechnol Lett* **22**, 12307-12315 (2017).

82 Valverde, E. E., Oster, A. M., Xu, S., Wertheim, J. O. & Hernandez, A. L. HIV Transmission Dynamics Among Foreign-Born Persons in the United States. *J Acquir Immune Defic Syndr* **76**, 445-452, doi:10.1097/QAI.0000000000001541 (2017).

83 Vanhommerig, J. W. *et al.* Limited overlap between phylogenetic HIV and hepatitis C virus clusters illustrates the dynamic sexual network structure of Dutch HIV-infected MSM. *Aids* **31**, 2147-2158, doi:10.1097/qad.0000000000001592 (2017).

84 Vrancken, B. *et al.* The multi-faceted dynamics of HIV-1 transmission in Northern Alberta: A combined analysis of virus genetic and public health data. *Infect Genet Evol* **52**, 100-105, doi:10.1016/j.meegid.2017.04.005 (2017).

85 Wang, X. *et al.* Phylodynamics of major CRF01_AE epidemic clusters circulating in mainland of China. *Scientific reports* **7**, 6330, doi:10.1038/s41598-017-06573-6 (2017).

86 Wertheim, J. O. *et al.* Social and Genetic Networks of HIV-1 Transmission in New York City. *PLoS Pathog* **13**, e1006000, doi:10.1371/journal.ppat.1006000 (2017).

87 Wolf, E. *et al.* Short Communication: Phylogenetic Evidence of HIV-1 Transmission Between Adult and Adolescent Men Who Have Sex with Men. *AIDS Res Hum Retroviruses* **33**, 318-322, doi:10.1089/aid.2016.0061 (2017).

88 Castley, A. S. *et al.* Longitudinal Trends in Western Australian HIV-1 Sequence Diversity and Viral Transmission Networks and Their Influence on Clinical Parameters: 2000-2014. *AIDS Res Hum Retroviruses* **32**, 211-219, doi:10.1089/aid.2015.0206 (2016).

89 Chin, B. S. *et al.* Molecular epidemiology identifies HIV transmission networks associated with younger age and heterosexual exposure among Korean individuals. *J Med Virol* **88**, 1832-1835, doi:10.1002/jmv.24523 (2016).
90 Esbjornsson, J. *et al.* HIV-1 transmission between MSM and heterosexuals, and increasing proportions of circulating recombinant forms in the Nordic Countries. *Virus Evol* **2**, vew010, doi:10.1093/ve/vew010 (2016).
91 Hoenigl, M. *et al.* Characterization of HIV Transmission in South-East Austria. *PLoS One* **11**, e0151478, doi:10.1371/journal.pone.0151478 (2016).
92 Junqueira, D. M., de Medeiros, R. M., Graf, T. & Almeida, S. E. Short-Term Dynamic and Local Epidemiological Trends in the South American HIV-1B Epidemic. *PLoS One* **11**, e0156712, doi:10.1371/journal.pone.0156712 (2016).
93 Konou, A. A. *et al.* Genetic diversity and transmission networks of HIV-1 strains among men having sex with men (MSM) in Lome, Togo. *Infect Genet Evol* **46**, 279-285, doi:10.1016/j.meegid.2016.05.030 (2016).
94 Magiorkinis, G. *et al.* The global spread of HIV-1 subtype B epidemic. *Infect Genet Evol* **46**, 169-179, doi:10.1016/j.meegid.2016.05.041 (2016).
95 Marzel, A. *et al.* HIV-1 Transmission During Recent Infection and During Treatment Interruptions as Major Drivers of New Infections in the Swiss HIV Cohort Study. *Clin Infect Dis* **62**, 115-122, doi:10.1093/cid/civ732 (2016).
96 Mehta, S. R. *et al.* Using HIV Sequence and Epidemiologic Data to Assess the Effect of Self-referral Testing for Acute HIV Infection on Incident Diagnoses in San Diego, California. *Clin Infect Dis* **63**, 101-107, doi:10.1093/cid/ciw161 (2016).
97 Panichsillapakit, T. *et al.* Prevalence of Transmitted HIV Drug Resistance Among Recently Infected Persons in San Diego, CA 1996-2013. *J Acquir Immune Defic Syndr* **71**, 228-236, doi:10.1097/QAI.0000000000000831 (2016).
98 Patino-Galindo, J. A. *et al.* Transmission dynamics of HIV-1 subtype B in the Basque Country, Spain. *Infect Genet Evol* **40**, 91-97, doi:10.1016/j.meegid.2016.02.028 (2016).
99 Pines, H. A. *et al.* Concurrency and HIV transmission network characteristics among MSM with recent HIV infection. *Aids* **30**, 2875-2883, doi:10.1097/qad.0000000000001256 (2016).
100 Poon, A. F. *et al.* Near real-time monitoring of HIV transmission hotspots from routine HIV genotyping: an implementation case study. *The lancet. HIV* **3**, e231-238, doi:10.1016/s2352-3018(16)00046-1 (2016).
101 Pouran Yousef, K. *et al.* Inferring HIV-1 Transmission Dynamics in Germany From Recently Transmitted Viruses. *J Acquir Immune Defic Syndr* **73**, 356-363, doi:10.1097/qai.0000000000001122 (2016).
102 Ragonnet-Cronin, M. *et al.* Transmission of Non-B HIV Subtypes in the United Kingdom Is Increasingly Driven by Large Non-Heterosexual Transmission Clusters. *J Infect Dis* **213**, 1410-1418, doi:10.1093/infdis/jiv758 (2016).
103 Ragonnet-Cronin, M. L. *et al.* A Direct Comparison of Two Densely Sampled HIV Epidemics: The UK and Switzerland. *Scientific reports* **6**, 32251, doi:10.1038/srep32251 (2016).
104 Shao, B. *et al.* Molecular epidemiology is becoming complex under the dynamic HIV prevalence: The perspective from Harbin, China. *J Med Virol* **88**, 807-814, doi:10.1002/jmv.24407 (2016).
105 Viciana, I. *et al.* Clinical, virological and phylogenetic characterization of a multiresistant HIV-1 strain outbreak in naive patients in southern Spain. *J Antimicrob Chemother* **71**, 357-361, doi:10.1093/jac/dkv332 (2016).
106 Wertheim, J. O. *et al.* The International Dimension of the U.S. HIV Transmission Network and Onward Transmission of HIV Recently Imported into the United States. *AIDS Res Hum Retroviruses* **32**, 1046-1053, doi:10.1089/aid.2015.0272 (2016).
107 Yebra, G. *et al.* Using nearly full-genome HIV sequence data improves phylogeny reconstruction in a simulated epidemic. *Scientific reports* **6**, 39489, doi:10.1038/srep39489 (2016).
108 Zeh, C. *et al.* Molecular Epidemiology and Transmission Dynamics of Recent and Long-Term HIV-1 Infections in Rural Western Kenya. *PLoS One* **11**, e0147436, doi:10.1371/journal.pone.0147436 (2016).

Supplementary Table S2. Difference of proportions of clustered sequences between method pairs using strict and relaxed sets of thresholds (support for Figure 3).

Methods comparison	Strict thresholds					Relaxed thresholds				
	Proportion 1	Proportion 2	Difference	Low 95% CI	High 95% CI	Proportion 1	Proportion 2	Difference	Low 95% CI	High 95% CI
PhyML vs HIV_TRACE	0.26	0.36	-0.09	-0.12	-0.06	0.54	0.36	0.18	0.15	0.22
FastTree_aLRT vs HIV_TRACE	0.27	0.36	-0.09	-0.12	-0.06	0.45	0.36	0.1	0.07	0.13
RAxML vs HIV_TRACE	0.27	0.36	-0.09	-0.12	-0.06	0.45	0.36	0.09	0.06	0.12
IQ-Tree_ufast vs HIV_TRACE	0.3	0.36	-0.05	-0.08	-0.02	0.48	0.36	0.13	0.1	0.16
FastTree_boot vs HIV_TRACE	0.24	0.36	-0.12	-0.15	-0.09	0.41	0.36	0.05	0.02	0.08
IQ-Tree_boot vs HIV_TRACE	0.23	0.36	-0.12	-0.15	-0.09	0.4	0.36	0.05	0.02	0.08
MEGA vs HIV_TRACE	0.22	0.36	-0.14	-0.17	-0.11	0.38	0.36	0.02	-0.01	0.05
PhyML vs IQ-Tree_boot	0.26	0.23	0.03	0	0.06	0.54	0.4	0.14	0.11	0.17
FastTree_boot vs PhyML	0.24	0.26	-0.02	-0.05	0	0.41	0.54	-0.13	-0.16	-0.1
PhyML vs MEGA	0.26	0.22	0.05	0.02	0.07	0.54	0.38	0.16	0.13	0.19
PhyML vs IQ-Tree_ufast	0.26	0.3	-0.04	-0.07	-0.01	0.54	0.48	0.06	0.02	0.09
RAxML vs PhyML	0.27	0.26	0	-0.03	0.03	0.45	0.54	-0.09	-0.12	-0.06
FastTree_aLRT vs PhyML	0.27	0.26	0	-0.03	0.03	0.45	0.54	-0.09	-0.12	-0.05
FastTree_aLRT vs MEGA	0.27	0.22	0.05	0.02	0.07	0.45	0.38	0.08	0.04	0.11
RAxML vs MEGA	0.27	0.22	0.05	0.02	0.07	0.45	0.38	0.07	0.04	0.1
RAxML vs IQ-Tree_boot	0.27	0.23	0.03	0	0.06	0.45	0.4	0.05	0.02	0.08
FastTree_aLRT vs IQ-Tree_boot	0.27	0.23	0.03	0	0.06	0.45	0.4	0.05	0.02	0.08
MEGA vs IQ-Tree_ufast	0.22	0.3	-0.09	-0.11	-0.06	0.38	0.48	-0.11	-0.14	-0.08
FastTree_boot vs IQ-Tree_ufast	0.24	0.3	-0.06	-0.09	-0.04	0.41	0.48	-0.07	-0.11	-0.04
RAxML vs FastTree_boot	0.27	0.24	0.03	0	0.05	0.45	0.41	0.04	0.01	0.07
FastTree_aLRT vs FastTree_boot	0.27	0.24	0.03	0	0.05	0.45	0.41	0.04	0.01	0.08
FastTree_aLRT vs IQ-Tree_ufast	0.27	0.3	-0.04	-0.07	-0.01	0.45	0.48	-0.03	-0.06	0
MEGA vs IQ-Tree_boot	0.22	0.23	-0.01	-0.04	0.01	0.38	0.4	-0.02	-0.06	0.01
FastTree_boot vs MEGA	0.24	0.22	0.02	-0.01	0.05	0.41	0.38	0.03	0	0.06
IQ-Tree_ufast vs IQ-Tree_boot	0.3	0.23	0.07	0.04	0.1	0.48	0.4	0.08	0.05	0.11
RAxML vs FastTree_aLRT	0.27	0.27	0	-0.03	0.03	0.45	0.45	0	-0.04	0.03
RAxML vs IQ-Tree_ufast	0.27	0.3	-0.04	-0.07	-0.01	0.45	0.48	-0.04	-0.07	0
FastTree_boot vs IQ-Tree_boot	0.24	0.23	0.01	-0.02	0.03	0.41	0.4	0.01	-0.02	0.04

Supplementary Table S3. Proportion of sequence pairs clustered by method pairs using strict set of thresholds (support for Figure 4A). The table shows proportions of sequence pairs identified in method A (at the top) that also clustered in method B (on the left).

Method	HIV-TRACE	IQ-Tree reg bootstrap	IQ-Tree ufast boot	MEGA	PhyML aLRT	FastTree reg bootstrap	FastTree aLRT	RAxML	Mean summary % concordance
RAxML	0.36	1.00	0.82	0.96	0.96	1.00	0.93	1.00	0.88
FastTree aLRT	0.38	0.98	0.85	0.92	0.97	0.98	1.00	0.97	0.86
FastTree reg bootstrap	0.35	0.98	0.78	0.90	0.93	1.00	0.90	0.96	0.87
PhyML aLRT	0.37	0.99	0.83	0.93	1.00	0.99	0.94	0.98	0.87
MEGA	0.17	0.42	0.38	1.00	0.43	0.44	0.41	0.45	0.66
IQ-Tree uf boot	0.41	0.99	1.00	0.98	0.99	0.99	0.99	0.99	0.83
IQ-Tree reg bootstrap	0.34	1.00	0.75	0.83	0.90	0.94	0.87	0.92	0.85
HIV-TRACE	1.00	0.97	0.90	0.97	0.97	0.98	0.97	0.97	0.65

Supplementary Table S4. Proportion of sequence pairs clustered by method pairs using relaxed set of thresholds (support for Figure 4B). The table shows proportions of sequence pairs identified in method A (at the top) that also clustered in method B (on the left).

Method	HIV-TRACE	IQ-Tree reg bootstrap	IQ-Tree ufast boot	MEGA	PhyML aLRT	FastTree reg bootstrap	FastTree aLRT	RAxML	Mean summary % concordance
RAxML	0.80	1.00	0.91	1.00	0.78	1.00	0.95	1.00	0.84
FastTree aLRT	0.80	0.99	0.93	0.99	0.69	1.00	1.00	0.86	0.86
FastTree reg bootstrap	0.79	1.00	0.82	0.99	0.61	1.00	0.86	0.78	0.87
PhyML aLRT	0.80	1.00	0.94	1.00	1.00	1.00	0.98	1.00	0.78
MEGA	0.48	0.61	0.50	1.00	0.38	0.61	0.53	0.48	0.73
IQ-Tree uf boot	0.80	1.00	1.00	0.99	0.69	1.00	0.97	0.86	0.85
IQ-Tree reg bootstrap	0.78	1.00	0.81	0.97	0.60	0.99	0.85	0.77	0.86
HIV-TRACE	1.00	0.73	0.61	0.72	0.45	0.73	0.64	0.57	0.69

Supplementary Table S5. Proportion of identical clusters in method pairs using strict set of thresholds (support for Figure 5A). The table shows proportions of clusters identified in method A (at the top) that are identical in method B (on the left).

Method	HIV-TRACE	IQ-Tree reg bootstrap	IQ-Tree ufast boot	MEGA	PhyML aLRT	FastTree reg bootstrap	FastTree aLRT	RAxML	Mean summary % concordance
RAxML	0.75	0.97	0.83	0.91	0.94	0.97	0.93	1.00	0.88
FastTree aLRT	0.74	0.93	0.81	0.85	0.94	0.94	1.00	0.91	0.87
FastTree reg bootstrap	0.67	0.91	0.72	0.79	0.85	1.00	0.84	0.85	0.85
PhyML aLRT	0.74	0.93	0.82	0.84	1.00	0.94	0.93	0.91	0.87
MEGA	0.70	0.84	0.74	1.00	0.81	0.85	0.81	0.85	0.82
IQ-Tree uf boot	0.77	0.92	1.00	0.88	0.94	0.92	0.93	0.93	0.83
IQ-Tree reg bootstrap	0.67	1.00	0.72	0.79	0.84	0.91	0.83	0.85	0.85
HIV-TRACE	1.00	0.79	0.71	0.78	0.78	0.79	0.78	0.77	0.75

Supplementary Table S6. Proportion of identical clusters in method pairs using relaxed set of thresholds (support for Figure 5B). The table shows proportions of clusters identified in method A (at the top) that are identical in method B (on the left).

Method	HIV-TRACE	IQ-Tree reg bootstrap	IQ-Tree ufast boot	MEGA	PhyML aLRT	FastTree reg bootstrap	FastTree aLRT	RAxML	Mean summary % concordance
RAxML	0.66	0.91	0.74	0.84	0.72	0.92	0.84	1.00	0.79
FastTree aLRT	0.58	0.78	0.72	0.72	0.65	0.81	1.00	0.81	0.73
FastTree reg. bootstrap	0.68	0.95	0.67	0.82	0.61	1.00	0.77	0.84	0.78
PhyML aLRT	0.52	0.72	0.63	0.65	1.00	0.71	0.71	0.76	0.64
MEGA	0.70	0.90	0.67	1.00	0.62	0.91	0.76	0.85	0.75
IQ-Tree ufast	0.59	0.76	1.00	0.69	0.62	0.77	0.79	0.78	0.68
IQ-Tree reg boot	0.69	1.00	0.65	0.80	0.61	0.94	0.73	0.82	0.78
HIV-TRACE	1.00	0.60	0.44	0.54	0.38	0.58	0.47	0.51	0.57

Supplementary Table S7. Proportion of non-clustered sequences in method pairs using strict set of thresholds (support for Figure 6A). The table shows proportions of non-clustered sequences in method A (at the top) that also did not cluster in method B (on the left).

Method	HIV-TRACE	IQ-Tree reg bootstrap	IQ-Tree ufast boot	MEGA	PhyML aLRT	FastTree reg bootstrap	FastTree aLRT	RAxML	Mean summary % concordance
RAxML	0.98	0.96	0.99	0.93	0.98	0.96	0.98	1.00	0.97
FastTree aLRT	0.98	0.95	0.99	0.92	0.98	0.96	1.00	0.98	0.96
FastTree reg bootstrap	0.99	0.98	1.00	0.94	0.99	1.00	0.99	1.00	0.96
PhyML aLRT	0.98	0.95	0.99	0.92	1.00	0.96	0.98	0.98	0.96
MEGA	0.99	0.96	1.00	1.00	0.98	0.96	0.98	0.99	0.94
IQ-Tree uf boot	0.97	0.90	1.00	0.89	0.94	0.91	0.94	0.94	0.95
IQ-Tree reg bootstrap	0.98	1.00	1.00	0.94	0.99	0.98	0.99	1.00	0.96
HIV-TRACE	1.00	0.83	0.90	0.81	0.86	0.84	0.86	0.86	0.92

Supplementary Table S8. Proportion of not clustered sequences in method pairs using relaxed set of thresholds (support for Figure 6B). The table shows proportions of non-clustered sequences in method A (at the top) that also did not cluster in method B (on the left).

Method	HIV-TRACE	IQ-Tree reg bootstrap	IQ-Tree ufast boot	MEGA	PhyML aLRT	FastTree reg bootstrap	FastTree aLRT	RAxML	Mean summary % concordance
RAxML	0.83	0.92	0.98	0.88	0.99	0.93	0.96	1.00	0.94
FastTree aLRT	0.83	0.90	0.97	0.85	0.98	0.91	1.00	0.95	0.93
FastTree reg bootstrap	0.88	0.98	0.99	0.92	1.00	1.00	0.99	1.00	0.94
PhyML aLRT	0.70	0.77	0.84	0.74	1.00	0.78	0.82	0.83	0.88
MEGA	0.87	0.96	0.98	1.00	1.00	0.97	0.97	1.00	0.91
IQ-Tree uf boot	0.79	0.85	1.00	0.81	0.94	0.86	0.92	0.91	0.92
IQ-Tree reg bootstrap	0.89	1.00	0.99	0.92	1.00	1.00	0.98	1.00	0.94
HIV-TRACE	1.00	0.95	0.99	0.90	0.99	0.96	0.97	0.97	0.89

Supplementary Figure Legends

Supplementary Figure S1. Summary characteristics of 108 reviewed papers using common analytical approaches for HIV-1 clustering. Bar graphs represent number or proportion (Y axis) of reviewed papers according to publication year (panel A), HIV-1 genes targeted for cluster analysis (B), sequencing methods (C), HIV-1 subtypes (D), Exclusion or retention of amino acid positions associated with HIV-1 drug resistance (E), and use of the ML model by model-based studies (F).

Supplementary Figure S2. Distribution of topological support and distance thresholds for identifying molecular HIV-1 clusters in the 108 reviewed papers. Bar graph represent the number of reviewed papers (Y axis) according to the distribution of topological support (panel A) and pairwise distance thresholds (panel B).

Supplementary Figure S3. Comparison of number of HIV clusters within commonly-used analytical approaches. See Figure 1 Legend for details.

Supplementary Figure S4. Comparison of numbers of identified HIV clusters between commonly-used analytical approaches. See Figure 2 Legend for details.

Figure S1

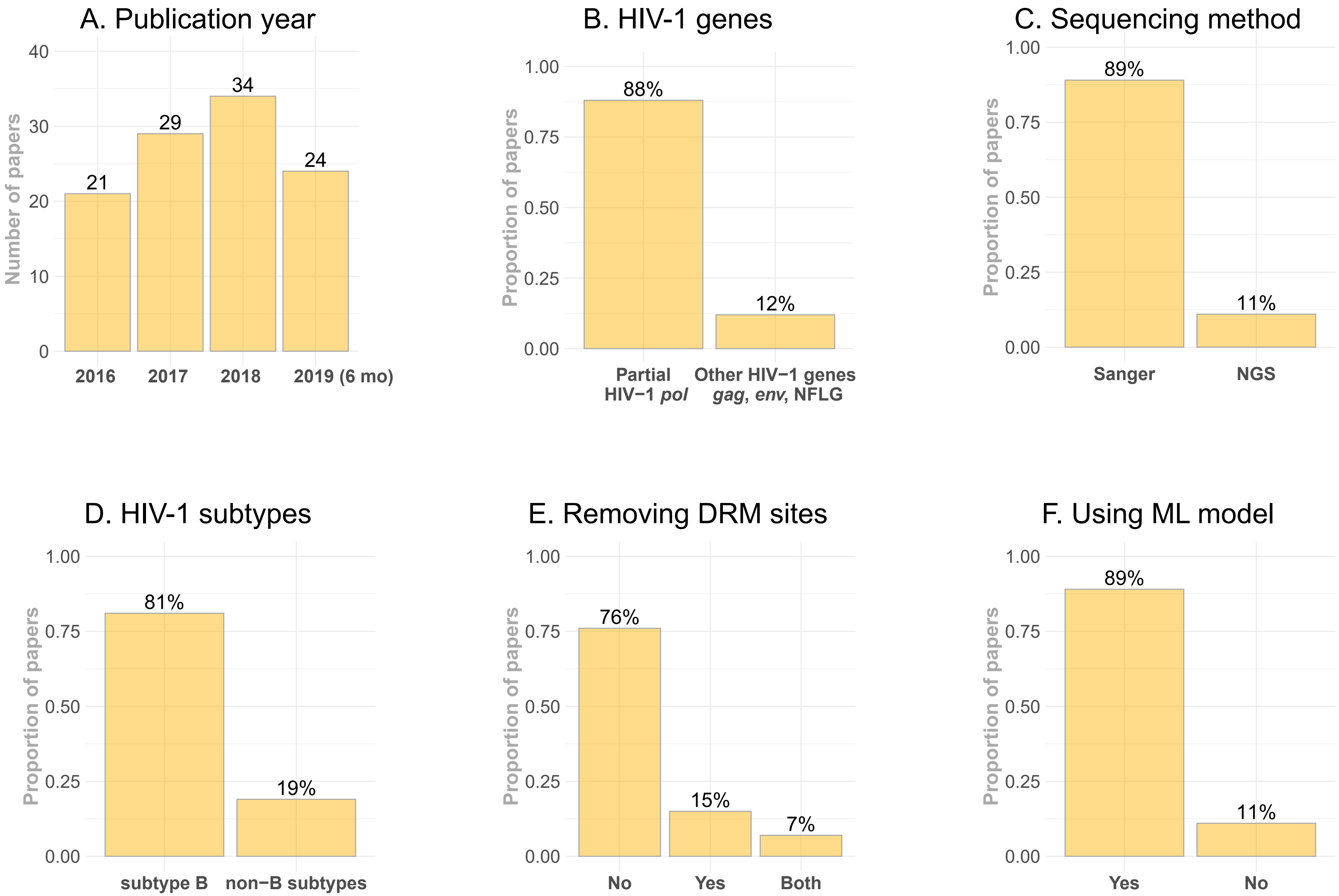

A. Publication year
Number of papers
0
10
20
30
40
21
29
34
24
2016
2017
2018
2019 (6 mo)
B. HIV-1 genes
Proportion of papers
0.00
0.25
0.50
0.75
1.00
88%
12%
Partial HIV−1 pol
Other HIV−1 genes gag, env, NFLG
C. Sequencing method
Proportion of papers
89%
11%
Sanger
NGS
D. HIV-1 subtypes
Proportion of papers
81%
19%
subtype B
non−B subtypes
E. Removing DRM sites
Proportion of papers
76%
15%
7%
No
Yes
Both
F. Using ML model
Proportion of papers
89%
11%
Yes
No

Figure S2

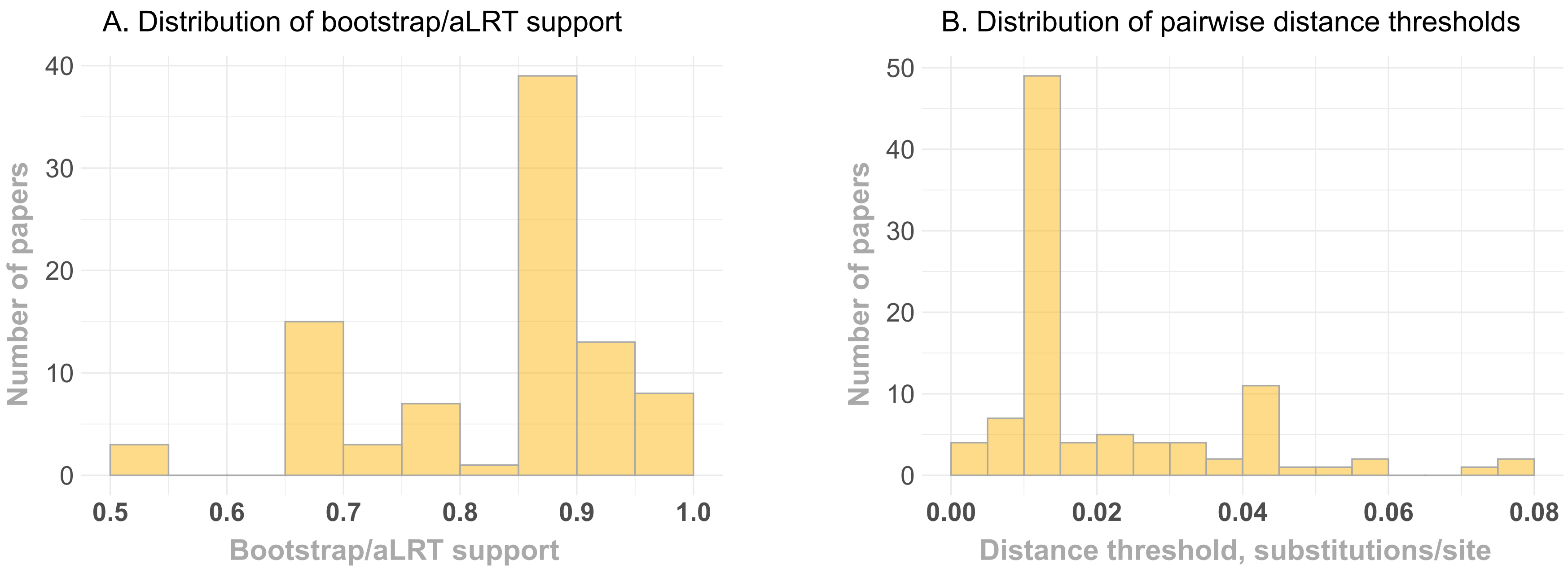

A. Distribution of bootstrap/aLRT support
Number of papers
0
10
20
30
40
0.5
0.6
0.7
0.8
0.9
1.0
Bootstrap/aLRT support
B. Distribution of pairwise distance thresholds
Number of papers
0
10
20
30
40
50
0.00
0.02
0.04
0.06
0.08
Distance threshold, substitutions/site

Figure S3

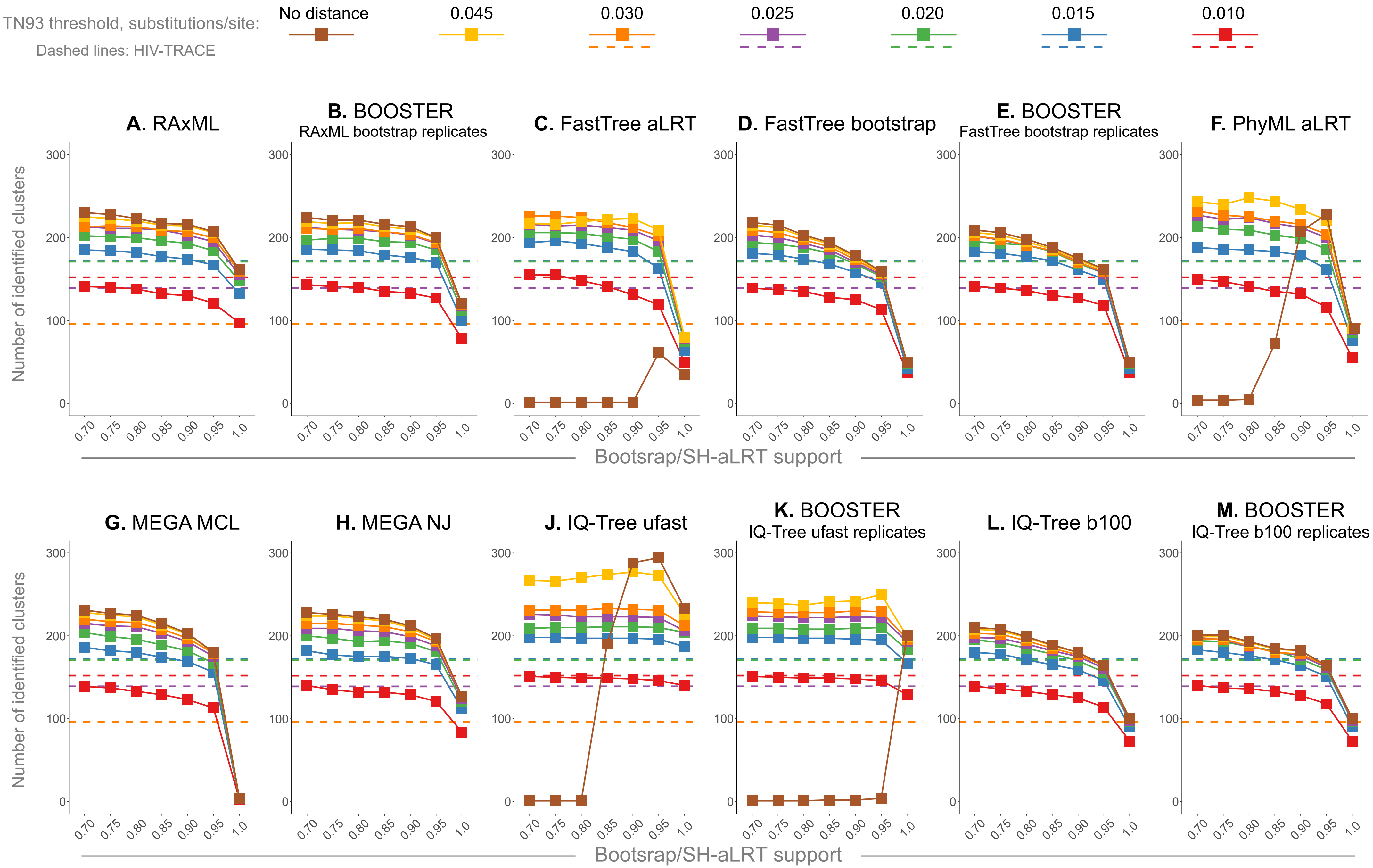
TN93 threshold, substitutions/site:
No distance
0.045
0.030
0.025
0.020
0.015
0.010
Dashed lines: HIV-TRACE
A. RAxML
B. BOOSTER
RAxML bootstrap replicates
C. FastTree aLRT
D. FastTree bootstrap
E. BOOSTER
FastTree bootstrap replicates
F. PhyML aLRT
G. MEGA MCL
H. MEGA NJ
J. IQ-Tree ufast
K. BOOSTER
IQ-Tree ufast replicates
L. IQ-Tree b100
M. BOOSTER
IQ-Tree b100 replicates
Number of identified clusters
0
100
200
300
0.70
0.75
0.80
0.85
0.90
0.95
1.0
Bootsrap/SH-aLRT support

Figure S4

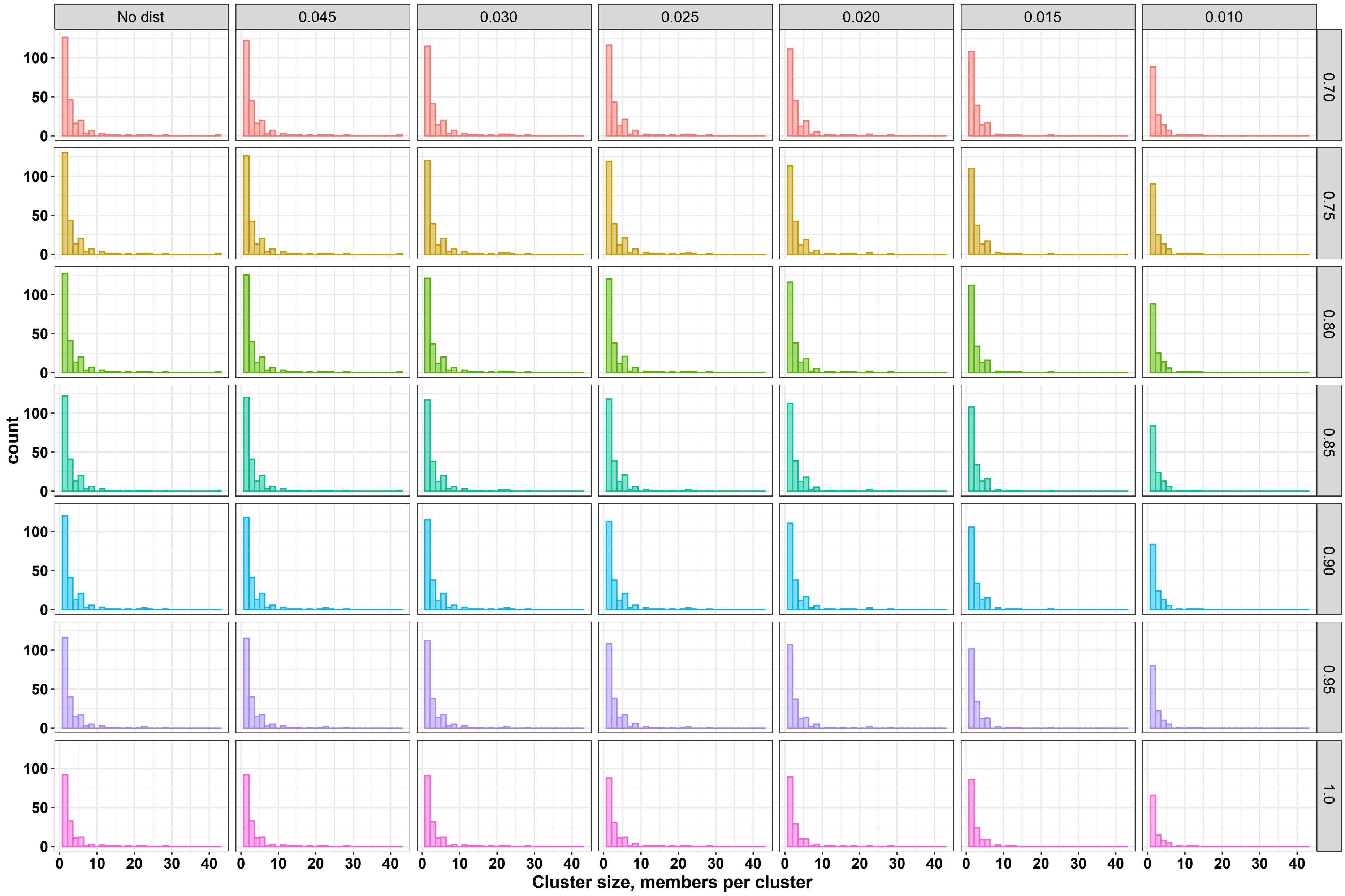

No dist
0.045
0.030
0.025
0.020
0.015
0.010
0.70
0.75
0.80
0.85
0.90
0.95
1.0
count
100
50
0
0
10
20
30
40
Cluster size, members per cluster

Figure S5

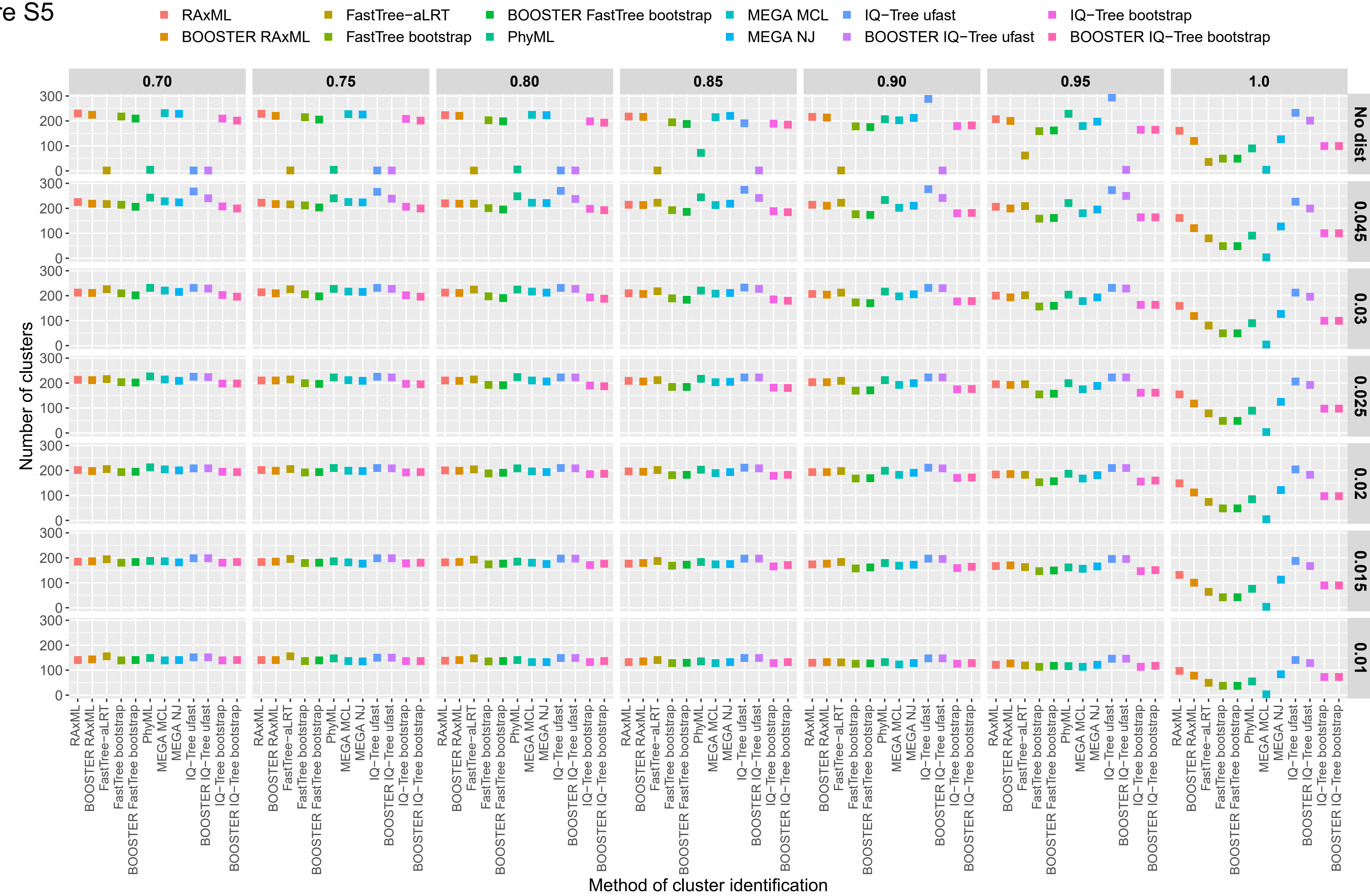
RAxML
BOOSTER RAxML
FastTree−aLRT
FastTree bootstrap
BOOSTER FastTree bootstrap
PhyML
MEGA MCL
MEGA NJ
IQ−Tree ufast
BOOSTER IQ−Tree ufast
IQ−Tree bootstrap
BOOSTER IQ−Tree bootstrap
0.70
0.75
0.80
0.85
0.90
0.95
1.0
No dist
0.045
0.03
0.025
0.02
0.015
0.01
Number of clusters
0
100
200
300
Method of cluster identification
